# Increased *TCP11* gene expression can inhibit the proliferation, migration and promote apoptosis of cervical cancer cells

**DOI:** 10.1186/s12885-023-11129-1

**Published:** 2023-09-11

**Authors:** Fang Wang, Shuyan Song, Bingxuan Guo, Yangyang Li, Huijuan Wang, Shaowei Fu, Luyue Wang, Xiangyi Zhe, Hongtao Li, Dongmei Li, Renfu Shao, Zemin Pan

**Affiliations:** 1 Department of Biochemistry and Molecular Biology, School of Medicine, Xinjiang Endemic and Ethnic Disease and Education Ministry Key Laboratory, Shihezi University, Shihezi, Xinjiang 832002 China; 2 Department of Clinical Laboratory, the First Affiliated Hospital of School of Medicine, Shihezi University, Shihezi, Xinjiang 832000 China; 3 Centre for Bioinnovation, School of Science, Technology and Engineering, University of the Sunshine Coast, Maroochydore, 4556 Australia; 4 Translational Medicine Center, Beijing Chest Hospital, Capital Medical University, Beijing, 101149 China

**Keywords:** Cervical cancer, *TCP11* gene, Cell cycle, Apoptosis, Migration and proliferation

## Abstract

**Background:**

Cervical cancer is a common gynecological malignancy. Gene microarray found that *TCP11* gene was highly expressed in cervical cancer. However, the effect of *TCP11* gene on the proliferation, apoptosis and migration of cervical cancer cells and its underlying molecular mechanisms are unclear.

**Methods:**

GEPIA database, tissue microarray, western blot and qRT-PCR were used to analyze the expression of *TCP11* gene in cervical cancer tissues and cells and its relationship with patients’ survival rate. The cell cycle and apoptosis were detected by flow cytometry, and the expressions of cell cycle and apoptosis related molecules and EMT-related molecules were detected by Western blot and qRT-PCR.

**Results:**

The results showed that *TCP11* gene was highly expressed in cervical cancer tissues and cells compared with normal cervical tissues and cells, and its expression was positively correlated with patients’ survival rate. The results of proliferation and migration assays showed that *TCP11* overexpression inhibited the proliferation and migration of HeLa and SiHa cells. The results showed that *TCP11 *overexpression blocked the cell cycle of HeLa and SiHa cells, decreased the expression of CDK1 and Cyclin B1, and increased the apoptosis and the expression of caspase-3, cleaved-caspase-3 and cleaved-PARP. *TCP11* overexpression increased the protein and mRNA expression of EMT-related molecules ZO-1 and E-cadherin. Conversely,* TCP11* knockdown promoted the proliferation of HeLa and SiHa cells and the migration of HeLa cells.

**Conclusions:**

*TCP11* overexpression significantly inhibited the occurrence and development of cervical cancer cells, it may be a potentially beneficial biomarker for cervical cancer.

**Supplementary Information:**

The online version contains supplementary material available at 10.1186/s12885-023-11129-1.

## Background

Cervical cancer is a common gynecological malignant disease in women [1–4]. The morbidity and mortality of cervical cancer have gradually increased in China, and it has gradually become younger [5]. In 2020, there were about 604,000 newly diagnosed cases and 342,000 deaths of cervical cancer worldwide [6]. Human papilloma virus (HPV) infection is a common sexually transmitted disease [7], and more than 90% of cervical cancer is caused by persistent high-risk HPV (HR-HPV) infection [8, 9]. Cervical cancer has no obvious symptoms in the early stage of infection, which is easy to be ignored and misdiagnosed. Moreover, cervical cancer has a high degree of malignancy and is prone to metastasis and invasion [10]. Although the diagnosis and treatment of cervical cancer has been improved, and more than 90% of patients with cervical cancer in the early stage of infection can be cured, the survival rate of patients with advanced cervical cancer is still very low, especially for patients with metastatic cervical cancer [11]. Tumor invasion and metastasis are still important obstacles to the treatment of cervical cancer [12].

According to PSORT (https://psort.hgc.jp/) analysis, TCP11 protein may be mainly distributed in the cytoplasm. TCP11 is evolutionarily conserved in most metazoans and contains an uncharacterized protein domain, the TCP11 domain, that comprises most of the protein [13]. *TCP11* gene is a human homologue of mouse *Tcp11* gene, which can encode fertilization-promoting peptide (FPP) receptor, and there are three splicing products named TCP11a, TCP11b and TCP11c [14]. It has been reported that TCP11 protein plays a role in sperm capacitation and acrosome reaction, and can regulate the activity of adenylate cyclase cAMP pathway [15]. In addition, changes in DNA methylation of *TCP11* gene are a characteristic of the sarcoma component of Uterine Carcinosarcoma (UCS) [16]. *TCP11* is overexpressed in bilateral varicocele patients, which can be used as a potential biomarker for bilateral varicocele [17].

At present, there is no article report on *TCP11* in cervical cancer. Interestingly, GEPIA database analysis showed that *TCP11* gene was highly expressed in cervical cancer, and its high expression was beneficial to the prognosis of cervical cancer patients. But, the effect of *TCP11* gene on the development of cervical cancer remains unclear. The purpose of this study was to evaluate the effect of *TCP11* on proliferation, apoptosis and migration of cervical cancer cells. The study revealed that *TCP11* may be a potential biomarker of cervical cancer, playing a role in the prevention, treatment and analysis of cervical cancer prognosis.

## Materials and methods

### The 22 K human genome array of CapitalBio

Four cases of cervical cancer and corresponding normal tissues adjacent to cancer were selected to extract tissue RNA by Trizol (Invitrogen, Gaithersburg, MD, USA) one-step method, and the total RNA was further purified by the NucleoSpin® RNA clean up kit (MACHEREY-NAGEL, Germany), quantified by spectrophotometer, and examined by formaldehyde denaturing gel electrophoresis. Fluorescent labeling of sample RNA (crystal core® CRNA amplification labeling kit). Hybridize and wash, and the labeled DNA is dissolved in 80 µL in the hybridization solution (3×SSC, 0.2% SDS, 5×Denhart’s, 25% formamide), hybridization at 42 ℃, overnight. After hybridization, 0.2% SDS, 2×Wash in liquid of SSC for 5 min, and then wash in 0.2×Wash in SSC at room temperature for 5 min. The slides can be used for scanning after being dried. The chip is scanned by LuxScan 10KA dual channel laser scanner (CapitalBio).

### Gene expression profiling interactive analysis (GEPIA) database

GEPIA database (http://gepia.cancer-pku.cn/index.html), developed by Peking University team, was used for gene expression analysis of 9736 tumors and 8587 normal samples from TCGA and GTEx databases [18]. In this paper, GEPIA database was used to analyze the expression of *TCP11* gene in cervical cancer and its relationship with survival rate.

### Sample Collection

The tissue wax blocks, Hematoxylin-Eosin (HE) sections and relevant medical history information of patients in the First Affiliated Hospital of School of Medicine, Shihezi University, Xinjiang from 2017 to 2019 were collected, including 31 normal cervical tissues and 35 cervical cancer tissues. The clinical information can be seen in supplementary Table S1. All specimens were diagnosed by two experienced pathologists, and all patients provided written informed consent. It has been approved by the Medical Ethics Committee of the First Affiliated Hospital of Shihezi University Medical School (Approval Number: KJ2020-051-01).

### Tissue microarray and immunohistochemistry

According to the HE sections of collected cervical tissue wax block, pathological experts marked the site of the cervical epithelium on the sections. Sampling was carried out according to the marker, and tissue microarray were made. The sections were deparaffinized with xylene and hydrated with alcohol, and then treated with citrate buffer for antigen-retrieval. After incubation in the dark in 3% H_2_O_2_, they were incubated with TCP11 antibody (Proteintech, China, 1:200) overnight. The next day, the sections were incubated with rabbit/mouse general secondary antibody (ZSGB-BIO, China) for 30 min. After DAB staining, the sections were stained with hematoxylin, dried and sealed, and the results were interpreted by pathological experts.

### Cell origin and cell culture

Cervical cancer SiHa cell line and HeLa cell line were purchased from Wuhan Boster Biological Technology Co., Ltd. in 2015. Cervical cancer C33A cell line and immortalized normal epithelial HaCaT cell line were purchased from Wuhan Procell Life Science&Technology Co., Ltd. in 2019. HaCaT cells were cultured on Dulbecco’s Modified Eagle Medium (DMEM) containing 15% Fetal Bovine Serum (FBS) and 1% penicillomycin, and cervical cancer cells were cultured on DMEM containing 10% FBS and 1% penicillomycin. The cells were cultured in a 37 °C incubator with 5% CO_2_, and passaged at intervals of 2–3 days.

### Cell transfection

The lentivirus overexpressing* TCP11* gene was designed and constructed by Shanghai Genechem Co., LTD. Lentiviruses were transfected in cervical cancer HeLa and SiHa cells. After 72 h of infection, puromycin (Solarbio, China) was used to select the stably transfected cells. The lentvirus that overexpresses *TCP11* gene is built with the transcript NM-001370687 (containing 503 amino acids) as the template. The selected GV341 vector is added with 3 Flag tags (each containing 8 amino acids), and the number of amino acids of TCP11 protein is 527. Therefore, after infection with lentivirus in cervical cancer cells, the molecular weight of TCP11 protein will increase, and the molecular weight size is about 58 kDa. The siRNA that interfered with *TCP11* gene was designed and constructed by Shanghai GenePharma Co., Ltd. Cervical cancer HeLa cells were transfected with siRNA using Lipofectamine 2000 (Invitrogen, USA) reagent. TCP11 siRNA sequence is as follows:

siRNA-TCP11-1: GCCUGAGAAUUGAGAUUGATT;

siRNA-TCP11-2: GCAGCCUAGUCUCCUUAAUTT;

siRNA-TCP11-3: GCUCUAAGCAGUGAUAAUATT;

siRNA-Negative Control: UUCUCCGAACGUGUCACGUTT.

### Western blot

Total cell protein was extracted by using cell lysate buffer (RIPA/ PMSF, Solarbio, China). The protein concentration was detected using BCA kit (Beyotime, China). Proteins were separated by SDS-PAGE electrophoresis, and transfered to PVDF membrane (Immobilon-P, USA). Then, the membrane strips were blocked with blocking solution (5% nonfat dry milk), cut according to different molecular weights of the target strips, and the primary antibody was incubated overnight at 4℃. After washing the membrane strips, the rabbit/mouse secondary antibody (ZSGB-BIO, China) was incubated at room temperature for 2 h. Finally, a chemiluminescence reagent (Thermo, USA) was added dropwise to the membrane strips, and a chemiluminescence instrument (Tanon, China) was used for exposure. Anti-GAPDH (TA-08) and anti-β-actin (TA-09) were purchased from ZSGB-BIO (China). Anti-TCP11 (14606-1-AP) and anti-caspase-3 (8193T) were purchased from Proteintech (China). Anti-cleaved-PARP (BSM-33,138 M), anti-CDK1 (BS-0542R), and anti-CyclinB1 (BS-0572R) were purchased from Bioss (China). Anti-ZO-1 (66470-2-IG), anti-Snail (C15D3), anti-Vimentin (D21H3), anti-β-catenin (D10A8), anti-Claudin-1 (D5H1D) and anti-E-cadherin (3195T) were purchased from Cell Signaling Technology (USA).

### Real-time quantitative PCR (qRT-PCR)

Trizol lysate buffer (Ambion, USA) was used to extract total cell RNA. Using the reverse transcription kit (TaKaRa, Japan), 1ug of each group of RNA was reversely transcribed into cDNA, and then PCR amplification was performed with GAPDH as internal reference. The amplification conditions were 95℃ for 30s, 95℃ for 3s, 60℃ for 30s, 40 cycles. The mRNA relative expression of target gene was calculated by 2^−ΔΔCt^ method. See Supplementary Table S2 for primer sequences.

### Thiazole blue (MTT) assay

Cervical cancer cells transfected with lentivirus or siRNA for 24 h were digested with trypsin and counted. Each well of 96-well plates was seeded with 1000 cells, and 5 replicates were set up for each group. MTT reagent (Solarbio, China) was added into wells, and incubated in the incubator for 3 h. The liquid in the wells was carefully discarded, and added with DMSO (MACKLIN, China). The 96-well plate was incubated on the shaker for 15 min in darkness. Cell proliferation was measured at 490 nm with BIO-RAD (USA) at 0, 24, 48, 72 and 96 h, respectively.

### Colony formation assay

Cervical cancer cells transfected with lentivirus or siRNA for 24 h were digested with trypsin and counted. Six-well plates were seeded with 500 cells/well. The culture was continued for about 2 weeks, and the medium was changed every 3–4 days. When the number of colony cells was > 50, or the cell colonies were visible to the naked eye, the culture was stopped. After washing the cells with PBS, they were fixed with 4% paraformaldehyde (Biosharp, China) for 30 min and stained with 0.1% crystal violet (Solarbio, China) for 30 min. Finally, the difference of colony number was analyzed by photographing and counting.

### Cell drop slice

Cervical cancer cells with stable infection of lentivirus were collected, fixed with 4% paraformaldehyde for 20 min and then incubated with 0.1% Triton X-100 (Solarbio, China) for 15 min to increase cell permeability. After the cells were washed with PBS, an appropriate amount of PBS was added for resuspension, and the trace suspension was absorbed and added to the 12-well slides soaked in poly-lysine, so that the cells presented a monolayer and a density of about 70%. After drying, 12-well slides were stored at -20℃ for using in subsequent experiment. After the slides were immunochemically stained (Ki67, ZSGB-BIO, 1:400), 4 areas were randomly selected, and Image Pro Plus software was used to measure the integrated optical density (IOD) value and area value. Mean density = IOD/area was calculated, which could reflect the protein expression level.

### Transwell transfer assay

Cell migration was measured using a 24-well plate with Transwell chambers (Corning Costar, USA). The cells were suspended in serum-free medium, and 200 µL cell suspension (100 cells/ml) was added into the upper chamber, and 600 µL DMEM medium containing 10% FBS was added into the lower chamber. After incubating for 36 h in an incubator at 37℃ and 5% CO_2_, they were fixed with 4% paraformaldehyde, stained with crystal violet, washed with water and dried. The number of transmembrane cells was counted in 9 fields of view (×400) randomly taken from each chamber membrane under a high magnification microscope.

### Cell scratch test

4 × 10^5^ cells transfected with lentivirus were collected and inoculated in 6-well plates for culture. When the cells have grown to about 80% of the cell density, the cells were scratched in a straight using 200 µL pipette tip perpendicular to the bottom. The inverted microscope was used to take pictures at 0, 24 and 48 h, trying to keep the same position.

### Cell cycle assay

Cervical cancer cells stably infected with lentivirus were collected and immobilized with pre-cooled 70% ethanol according to the instructions of the Cell Cycle Detection Kit (LIANKE, China). On the day of detection, 1mL DNA Staining Solution was added and let stand for 30 min avoiding light at room temperature. The cell cycle distribution was detected and analyzed by flow cytometry, and > 10,000 cells were collected per sample.

### Cell apoptosis assay

Cervical cancer cells stably infected with lentivirus were collected. According to the instructions of apoptosis Detection Kit (LIANKE, China), apoptosis was detected by flow cytometry using Annexin V-FITC and PI double staining.

### Statistical analysis

All data input was organized by Excel software, processed and analyzed by SPSS 22.0. Statistics of measurement data were expressed as ${\bar{{\rm X}}}$ ± s, differences between groups were tested by* t *test, and tissue microarray results were tested by χ2 test. *P* < 0.05 was considered to be statistically significant.

## Results

### TCP11 is highly expressed in cervical cancer tissues and cells, which is closely related to the survival rate of cervical cancer patients

**Fig. 1:**
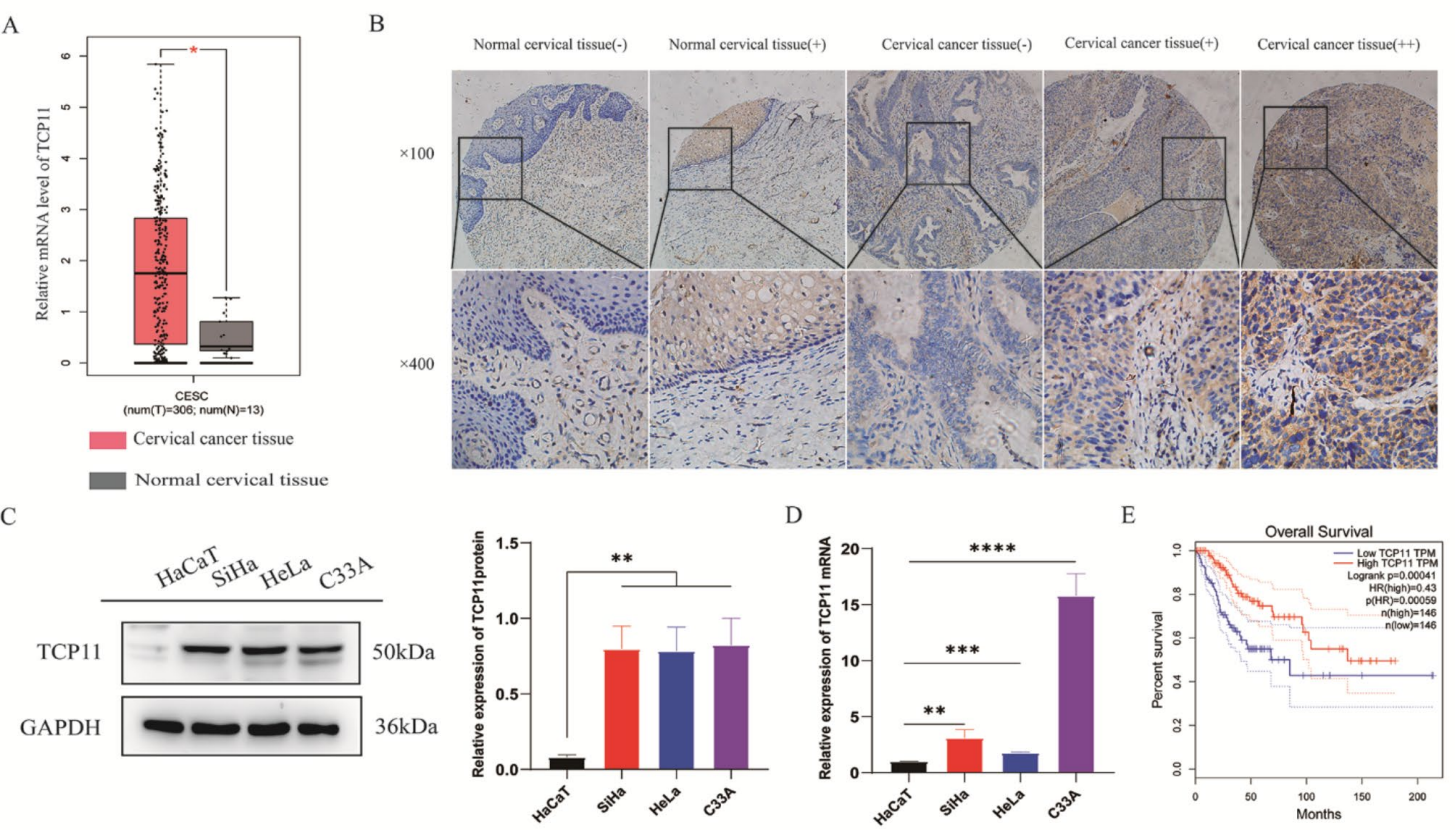
*TCP11* gene is highly expressed in cervical cancer tissues and cells, and is closely associated with patients’ survival rate. **A**: The mRNA expression of *TCP11* in normal cervical tissues and cervical cancer tissues were analyzed by GEPIA database. **B**: Immunohistochemical results of TCP11 protein expression in normal cervical tissues (n = 31) and cervical cancer tissues (n = 35) microarray. “-” indicates that the expression of TCP11 is negative; “+” indicates that the expression of TCP11 is weakly positive; “++” indicates that the expression of TCP11 is medium positive. Magnification: ×100 (top) and ×400 (bottom). **C**: Western blot was used to detect the expression of TCP11 protein in immortalized epithelial cells HaCaT and three cervical cancer cells. Full-length blots/gels are presented in Supplementary Figure S1. **D**: qRT-PCR was used to detect the expression of *TCP11* mRNA in immortalized epithelial cells HaCaT and three cervical cancer cells. **E**: GEPIA database was used to analyze the relationship between the expression of *TCP11* and survival rate of cervical cancer patients. Data are presented as mean ± SD of at least 3 independent experiments. *P < 0.05, **P < 0.01, ***P < 0.001, ****P < 0.0001

In the early stage, the research group collected 4 cases of cervical cancer and corresponding adjacent normal tissues to make a gene microarray (The 22 K Human Genome Array of CapitalBio) to screen differentially expressed genes. The previous gene microarray results found that the expression of *TCP11* gene in cervical cancer tissue was significantly higher than that in normal cervical tissue (Table 1). However, *TCP11* gene was not reported in cervical cancer, which aroused our research interest. To initially explore the role of *TCP11* in cervical cancer, we used GEPIA database for predictive analysis. The results showed that the mRNA expression of *TCP11* in cervical cancer tissues was higher than that in normal cervical tissues (Fig. 1A). Immunohistochemical staining was performed on the tissue to confirm the predicted results. The expression of TCP11 protein in cervical cancer tissues was higher than that in normal cervical tissues. In 31 cases of normal cervical tissue, the weak positive rate of TCP11 expression was 48.39%, and the negative rate was 51.61%; in 35 cases of cervical cancer tissue, the positive rate of TCP11 expression was 14.29%, the weak positive rate was 57.14%, and the negative rate was 28.57% (Fig. 1B; Table 2). In addition, western blot and qRT-PCR were used to detect the expression of TCP11 protein and mRNA in immortalized epithelial cells HaCaT and cervical cancer HeLa, SiHa and C33A cells, respectively. Compared with immortalized epithelial HaCaT cells, TCP11 protein and mRNA were highly expressed in three cervical cancer cell lines (Fig. 1C and D). Interestingly, based on the GEPIA database, we found that cervical cancer patients with high expression of *TCP11* had significantly higher overall survival than those with low expression (Fig. 1E). These results indicate that *TCP11* gene may play an important role in the development of cervical cancer.

**Table 1:** The statistical results of *TCP11* gene expression were analyzed by gene expression chip

Name	Mean	1 + 2	3 + 4	3CA + 3CA side	5CA + 5CA side
TCP11	6.3800	3.6360	5.9840	3.0817	12.8181

The “Mean” value greater than 2.0 indicates that the gene is highly expressed in cancer tissues. “1” represents cervical cancer tissue, and “2” represents corresponding adjacent tissue of cervical cancer; “3” represents cervical cancer tissue, and “4” represents cervical cancer corresponding adjacent tissue; “3CA” and “3CA side” represent cervical cancer and its corresponding adjacent tissues respectively; “5CA” and “5CA side” represent cervical cancer and its corresponding adjacent tissues respectively.

**Table 2:** Statistical table of immunohistochemical results of TCP11 in normal cervix and cervical cancer tissues

Sample	-	+	++	Total	*P* value
Normal cervical tissue	16	15	0	31	**0.019**
Cervical cancer tissue	10	20	5	35	

“-” indicates that the expression of TCP11 is negative; “+” indicates that the expression of TCP11 is weakly positive; “++” indicates that the expression of TCP11 is medium positive

### *TCP11* overexpression inhibits the proliferation of cervical cancer cells

**Fig. 2:**
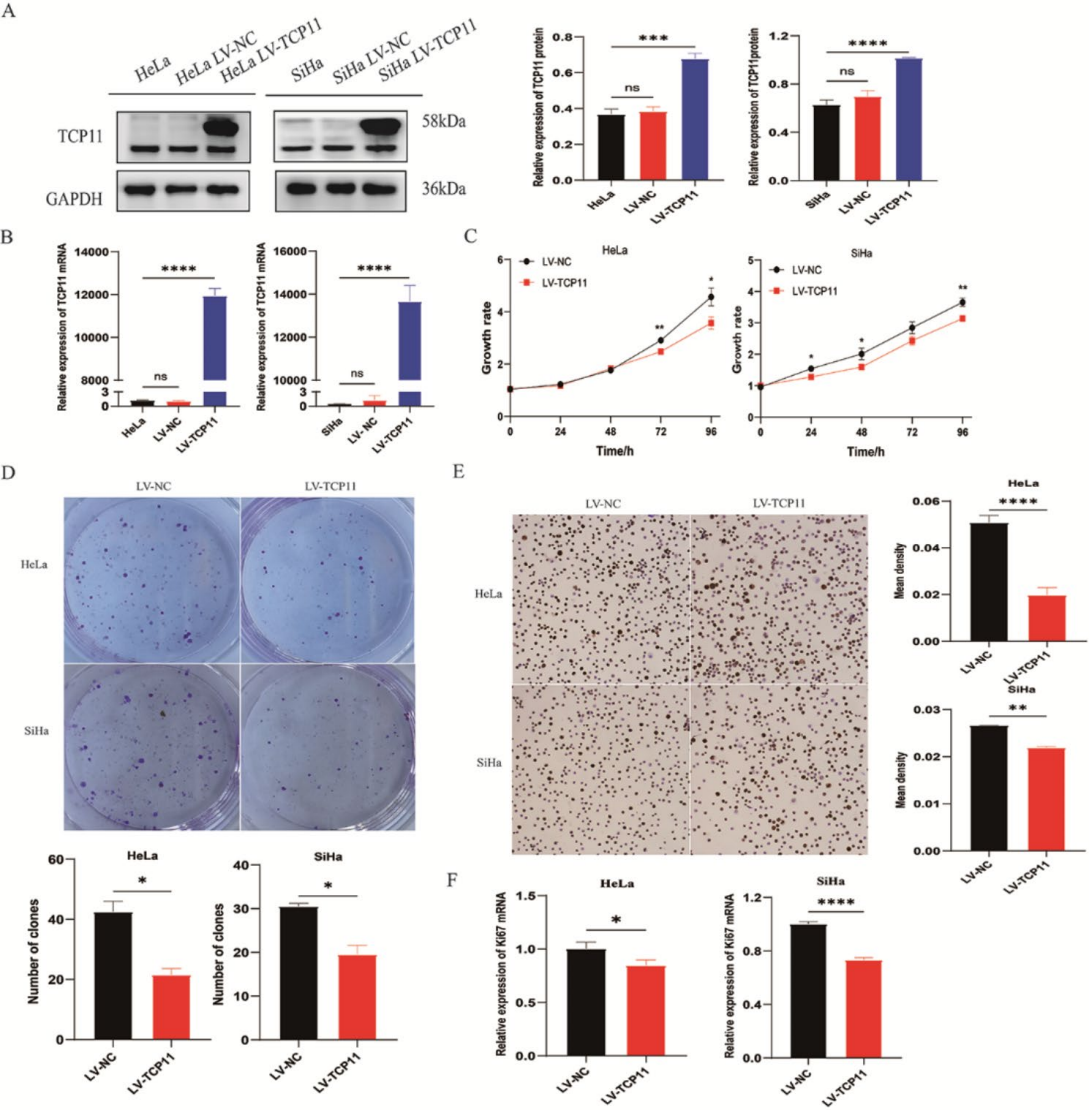
*TCP11* overexpression inhibits the proliferation of HeLa and SiHa cells. **A**: Western blot was used to detect the overexpression of TCP11 protein in HeLa and SiHa cells after lentivirus infection. Full-length blots/gels are presented in Supplementary Figure S2. **B**: qRT-PCR was used to detect the overexpression of *TCP11* mRNA in HeLa and SiHa cells after lentivirus infection. **C** and **D**: The cell viability of HeLa and SiHa cells was detected by MTT and colony formation assays. **E**: The protein expression of Ki67 in HeLa and SiHa cells was detected by cellular immunohistochemistry. Magnification: ×100. The results were analyzed by Image Pro Plus software, and the mean density was calculated (mean density = IOD/area). **F**: Ki67 mRNA expression in HeLa and SiHa cells were detected by qRT-PCR. Data are presented as mean ± SD of at least 3 independent experiments. *P < 0.05, **P < 0.01, ****P < 0.0001

To confirm the role of *TCP11* in cervical cancer, we first investigated whether overexpression of *TCP11* by lentivirus infection had an effect on the proliferation of cervical cancer cells. After lentivirus infection with cervical cancer HeLa and SiHa cells, the expression of TCP11 protein and mRNA was detected to confirm the successful selection of stably transfected cells overexpressing *TCP11 *(Fig. 2A and B). MTT assay showed that *TCP11 *overexpression significantly inhibited the cell viability of HeLa and SiHa cells (Fig. 2C). This result was further confirmed by the colony formation assay. Compared with the NC group, the number of colony formation in the overexpression group was significantly reduced (Fig. 2D). In addition, we analyzed the protein and mRNA expression of the proliferation marker Ki67 by using cellular immunohistochemistry and qRT-PCR. *TCP11* overexpression significantly inhibited the expression of Ki67 in cervical cancer cells (Fig. 2E and F). These results suggest that* TCP11* plays an important role in inhibiting the proliferation of cervical cancer cells.

### *TCP11* overexpression blocks the cell cycle progression of cervical cancer cells

**Fig. 3:**
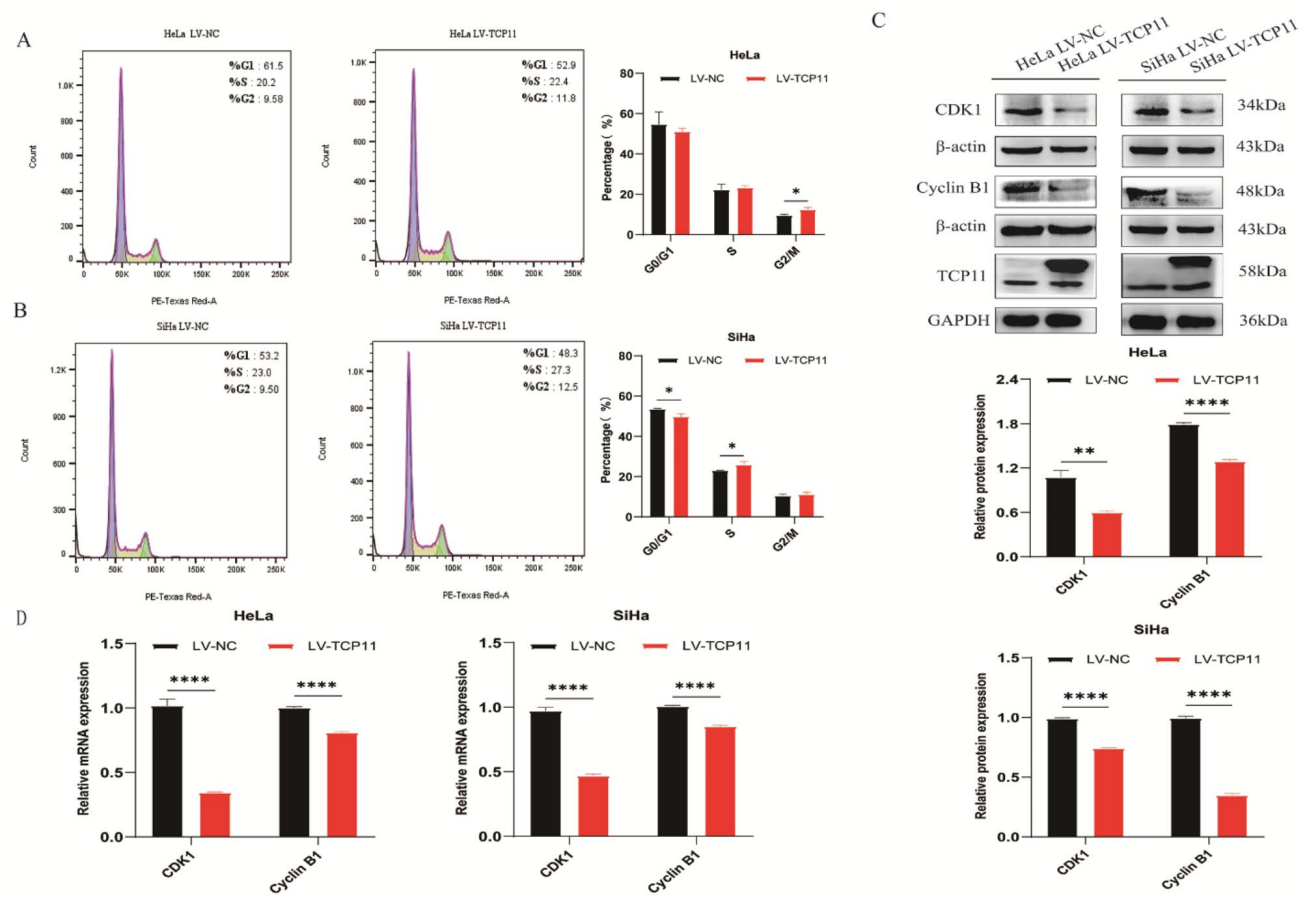
*TCP11* overexpression blocks cell cycle progression in HeLa and SiHa cells. **A** and **B**: Cell cycle distribution of HeLa and SiHa cells was determined by flow cytometry. **C**: Western blot was used to detect the protein expression of CDK1 and Cyclin B1 in HeLa and SiHa cells infected with overexpressing TCP11 lentivirus. Full-length blots/gels are presented in Supplementary Figure S3. **D**: qRT-PCR was used to detect the mRNA expression of CDK1 and Cyclin B1 in HeLa and SiHa cells infected with overexpressing TCP11 lentivirus. Data are presented as mean ± SD of at least 3 independent experiments. *P < 0.05, **P < 0.01, ****P < 0.0001

To investigate the reason why *TCP11* inhibits the proliferation of cervical cancer cells, flow cytometry was used to analyze the effect of* TCP11* overexpression on cell cycle progression. The results showed that the number of HeLa cells in G2/M phase in the overexpressed group increased compared with the NC group (Fig. 3A). The number of SiHa cells in S phase increased, and the number of SiHa cells in G2/M phase also increased, but there was no statistical significance (Fig. 3B).

To further explore the potential reasons of *TCP11* blocking cell cycle, western blot and qRT-PCR were used to detect the expression changes of cell cycle-related molecules CDK1 and Cyclin B1. After overexpression of *TCP11*, the protein and mRNA expressions of CDK1 and Cyclin B1 were decreased in HeLa and SiHa cells (Fig. 3C and D). The above results indicate that *TCP11* may block the cell cycle by inhibiting the expression of CDK1/Cyclin B1, thereby inhibiting the proliferation of cervical cancer cells.

### *TCP11* overexpression promotes apoptosis of cervical cancer cells

**Fig. 4:**
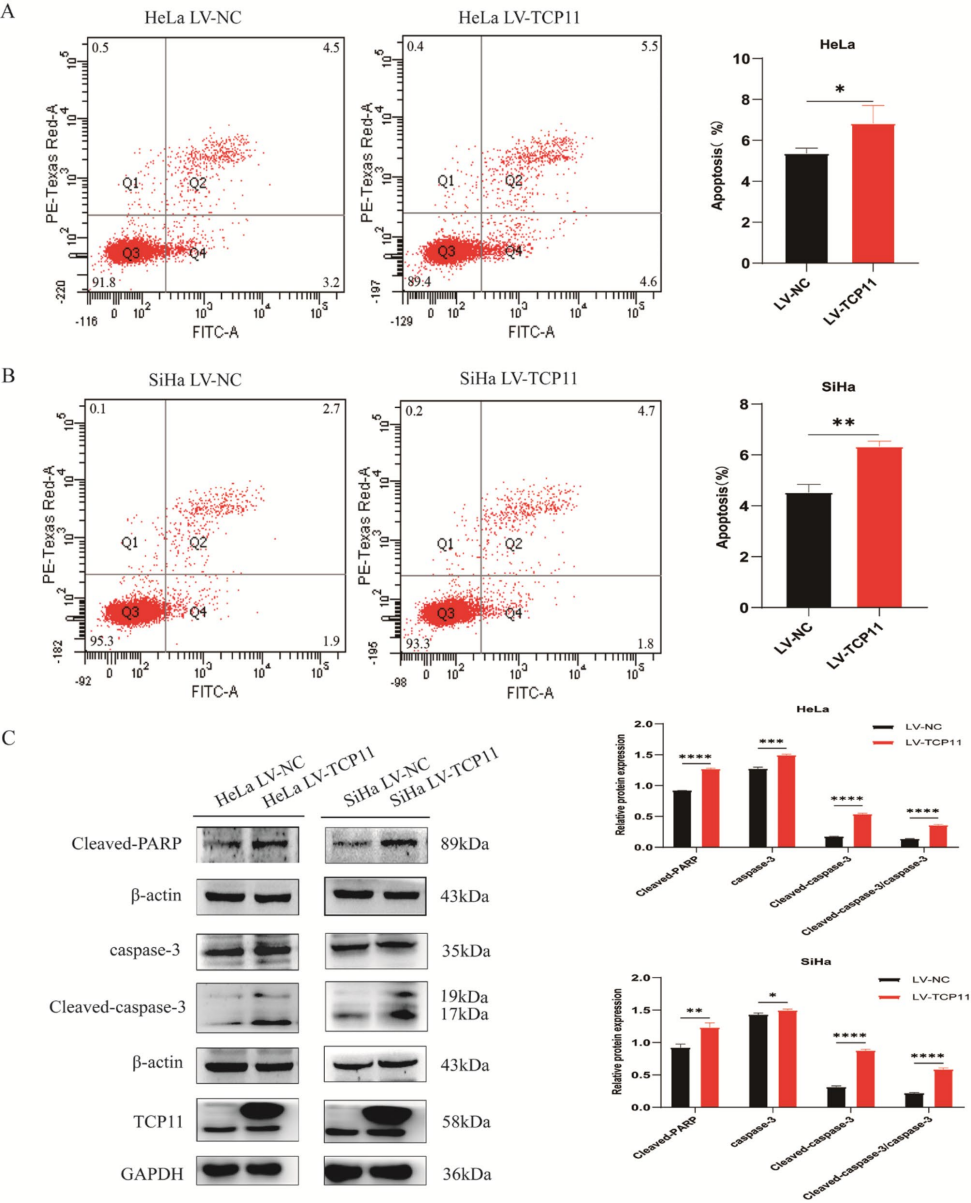
*TCP11* overexpression induces the apoptosis of HeLa and SiHa cells. **A** and **B**: Apoptosis of HeLa and SiHa cells were detected by flow cytometry. **C**: Western blot was used to detect the protein expression of caspase-3, cleaved-caspase-3 and cleaved-PARP in HeLa and SiHa cells infected with overexpressing TCP11 lentivirus. Full-length blots/gels are presented in Supplementary Figure S4. Data are presented as mean ± SD of at least 3 independent experiments. *P < 0.05, **P < 0.01, ***P < 0.001, ****P < 0.0001

Flow cytometry was used to analyze whether the expression of *TCP11* has an effect on the apoptosis of cervical cancer cells. The results showed that the apoptosis of HeLa and SiHa cells increased in the overexpressed group compared with the NC group (Fig. 4A and B). In order to better understand the potential reasons of TCP11 promoting apoptosis of cervical cancer cells, we used western blot to analyze the protein expression of apoptosis-related molecules caspase-3, cleaved-caspase-3 and cleaved-PARP. The results showed that *TCP11* overexpression significantly increased the expression of caspase-3, cleaved-caspase-3 and cleaved-PARP (Fig. 4C).

### *TCP11* overexpression inhibits cervical cancer cell migration by inhibiting EMT of cervical cancer cells

**Fig. 5:**
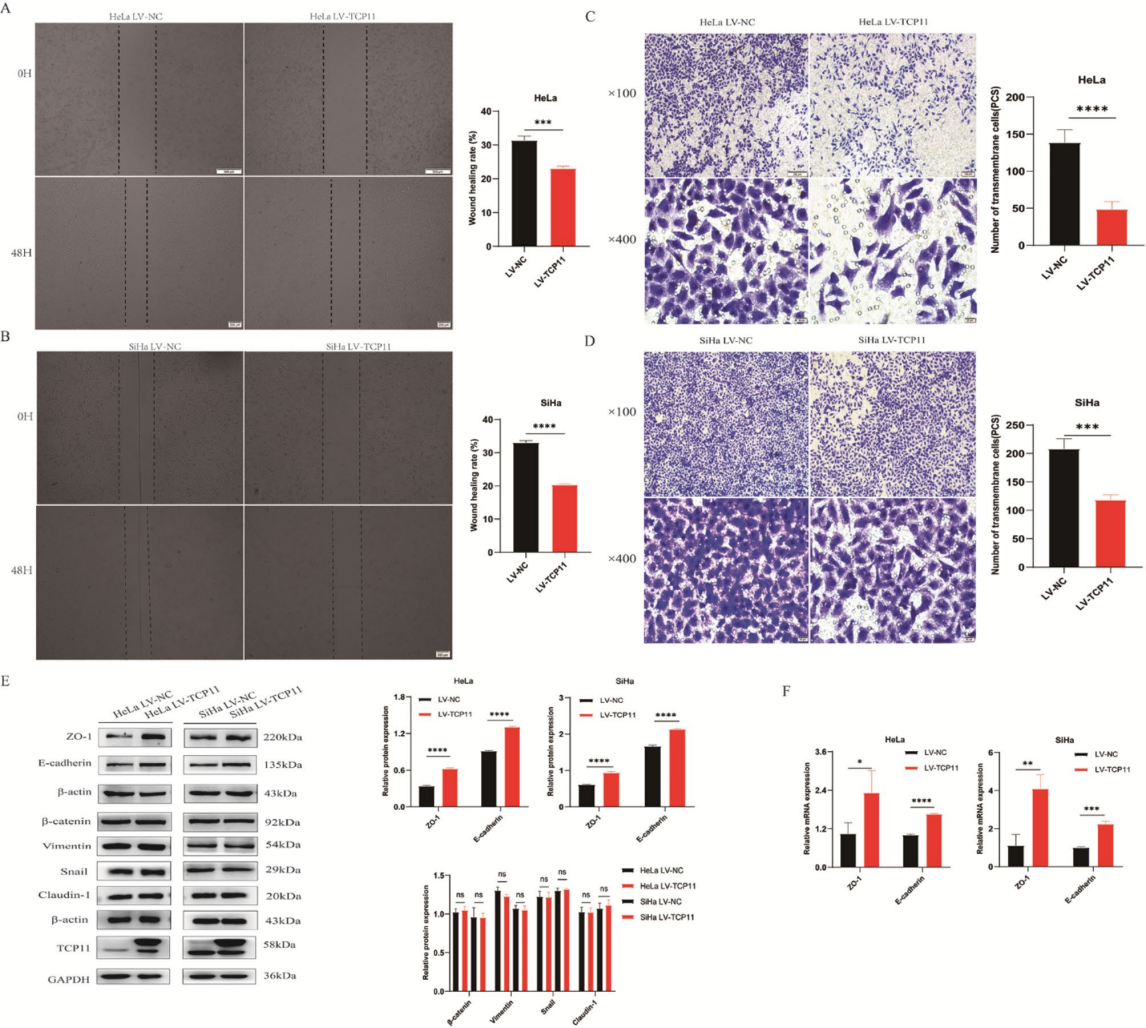
*TCP11* overexpression inhibits cervical cancer cell migration by inhibiting EMT of HeLa and SiHa cells. **A** and **B**: Cell scratch assay was used to detect the migration ability of HeLa and SiHa cells. **C** and **D**: Transwell migration assay was used to detect the migration ability of HeLa and SiHa cells. **E**: Western blot was used to detect the protein expression of EMT-related molecules ZO-1, E-cadherin, Claudin-1, Snail, Vimentin and β-catenin in HeLa and SiHa cells infected with overexpressing TCP11 lentivirus. Full-length blots/gels are presented in Supplementary Figure S5. **F**: qRT-PCR was used to detect the mRNA expression of EMT-related molecules ZO-1 and E-cadherin in HeLa and SiHa cells infected with overexpressing TCP11 lentivirus. Data are presented as mean ± SD of at least 3 independent experiments. *P < 0.05, **P < 0.01, ***P < 0.001, ****P < 0.0001

Migration is one of the key steps in tumor cells metastasis. Inhibition of cell migration can effectively prevent tumor progression. Cell scratch assay showed that *TCP11* overexpression significantly inhibited the scratch healing rate of HeLa and SiHa cells (Fig. 5A and B). In addition, transwell migration assay showed that the number of transmembrane cells of HeLa and SiHa cells in the overexpressed group was significantly lower than that in the NC group (Fig. 5C and D). These results indicate that *TCP11* plays an important role in inhibiting cell migration.

EMT is the key to the migration and invasion of malignant tumor cells, and the process of EMT plays an important role in the development of cervical cancer. Analysis of the expression changes of EMT-related molecules could reveal the potential reasons of *TCP11* gene affecting cervical cancer cell migration. Our results showed that the protein and mRNA expressions of ZO-1 and E-cadherin were significantly increased after overexpression of TCP11 in HeLa and SiHa cells, but Claudin-1, Snail, Vimentin and β-catenin showed no significant difference (Fig. 5E and F). This suggests that *TCP11* may inhibit the migration of cervical cancer cells by increasing tight junctions and adhesion between cells.

### *TCP11* knockdown promotes the proliferation and migration of cervical cancer HeLa cells

**Fig. 6:**
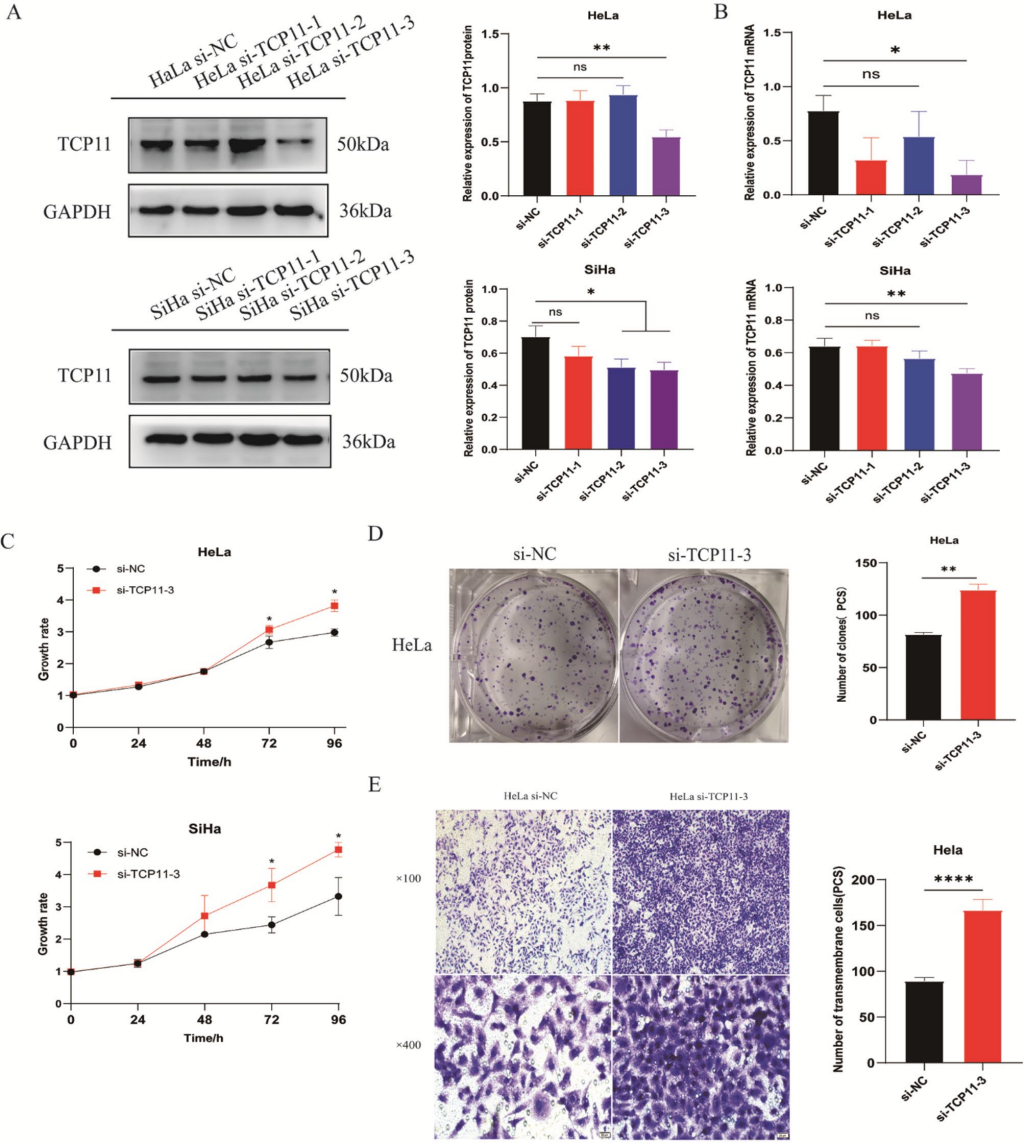
*TCP11* knockdown promotes the proliferation of HeLa and SiHa cells and the migration of HeLa cells. **A**: Western blot was used to detect the knockdown effect of three designed and constructed siRNA fragments on TCP11 in HeLa and SiHa cells. Full-length blots/gels are presented in Supplementary Figure S6. **B**: qRT-PCR was used to detect the knockdown effect of three siRNA fragments on *TCP11 *in HeLa and SiHa cells. **C**: MTT assay was used to detect the effect of *TCP11* knockdown on the proliferation of HeLa and SiHa cells. **D**: Colony formation assay was used to detect the effect of *TCP11* knockdown on the proliferation of HeLa cells. **E**: Transwell migration assay was used to detect the effect of *TCP11* knockdown on the migration of HeLa cells. Data are presented as mean ± SD of at least 3 independent experiments. *P < 0.05, **P < 0.01, ****P < 0.0001

To further confirm the inhibitory effect of *TCP11* on the proliferation and migration of cervical cancer cells, three siRNA fragments targeting different positions were transfected into cervical cancer HeLa and SiHa cells, and it was found that si-TCP11-3 successfully knocked down the expression of TCP11 in HeLa and SiHa cells (Fig. 6A and B). MTT assay showed that the cell viability of HeLa and SiHa cells in the knockdown group was significantly higher than that in the NC group (Fig. 6C). Colony formation assay also showed that* TCP11* knockdown increased the colony number of HeLa cells (Fig. 6D). Transwell migration assay showed that *TCP11* knockdown significantly increased the mobility of HeLa cells (Fig. 6E).

## Discussion

According to Uniprot database (https://www.uniprot.org/), *TCP11* gene is related to cell differentiation, signal transduction, multicellular biological development and protein kinase A signal transduction. In this study, through GEPIA database and tissue microarray assay, we demonstrated that the expression of *TCP11* gene was higher in cervical cancer tissues than in normal cervical tissues. It is worth noting that the* TCP11* had low expression in testicular germ cell tumor in GEPIA database, which was contrary to the high expression in cervical cancer. This may be because *TCP11* gene is a testicle-specific gene product and enriched in normal testicle, but the expression of *TCP11* is reduced when the testis becomes cancerous, for reasons that are not clear. Interestingly, cervical cancer patients with high expression of *TCP11* had higher survival rates. This suggests that *TCP11* may be a prognostic indicator for cervical cancer patients. Furthermore, we found that *TCP11* overexpression significantly inhibited the proliferation and migration of cervical cancer HeLa and SiHa cells. Conversely, the low expression of *TCP11* promoted the cell proliferation in HeLa and SiHa cells. Knocking down the expression level of *TCP11* gene can promote the cell migration in HeLa cells. *TCP11* gene can affect the proliferation and migration of cervical cancer cells, possibly by affecting the expression of cell cycle related molecules and EMT related molecules. Therefore, we next analyzed the characteristics of the *TCP11* gene acting on these molecules.

It has been shown that the disorder of cell cycle regulation is one of the main factors leading to the proliferation of malignant cells [19]. We found that overexpression of *TCP11* arrested the cell cycle of cervical cancer cells, HeLa cells were arrested in G2/M phase, SiHa cells were arrested in S phase. Cyclin B1 is a cyclin-related protein of the M phase, which can bind to CDK1 and activate CDK1 to realize the cell cycle transition from G2 phase to M phase [20, 21]. The results showed that *TCP11* overexpression inhibited the protein and mRNA expressions of CDK1 and Cyclin B1. We speculate that *TCP11* gene may block the cell cycle by regulating the expression of CDK1/Cyclin B1, thereby inhibiting the proliferation of cervical cancer cells. In addition, we also explored the effect of *TCP11* on apoptosis of cervical cancer cells. *TCP11* gene may induce cell apoptosis by activating caspase-3, further activating its substrate PARP. EMT is the key to migration and invasion of malignant tumor cells [22, 23]. To further investigate whether *TCP11* inhibits the migration of cervical cancer cells by regulating EMT, we detected the protein and mRNA expressions of EMT-related molecules ZO-1 and E-cadherin. The results showed that *TCP11* overexpression can inhibit the EMT process by increasing the protein and mRNA expressions of ZO-1 and E-cadherin, thereby inhibiting the migration of cervical cancer cells. Further research is needed on the proliferation and migration effects of *TCP11* gene on cervical cancer cells.

According to UniProt database analysis, *TCP11 *gene can regulate the activity of cAMP pathway of adenylyl cyclase. cAMP as a second messenger can activate cAMP-dependent protein kinase (PKA) [24, 25]. cAMP-PKA signaling pathway can regulate many cellular responses, including proliferation, apoptosis, migration and metabolism [26–28]. It has been shown that cAMP/PKA signaling pathway can inhibit the migration of cervical cancer HeLa cells [29]. *TCP11* has been reported to be correlated with PKA signal transduction [30]. Therefore, we speculated whether *TCP11 *gene could inhibit the progression of cervical cancer cells by regulating the activity of cAMP/PKA signaling pathway. However, further studies are needed to verify this hypothesis.

## Conclusions

In summary, our results suggest that *TCP11* plays an important role in the development of cervical cancer. *TCP11* overexpression can inhibit the proliferation and migration of cervical cancer cells, block cell cycle and induce apoptosis. In addition, *TCP11* overexpression inhibited cell migration by inhibiting EMT-related molecules. *TCP11* may be a potentially beneficial biomarker for cervical cancer. However, we still need to further study which specific signaling pathway can regulate the inhibitory effect of *TCP11* on cervical cancer progression.

### Electronic supplementary material

Below is the link to the electronic supplementary material.

Supplementary Material 1

## Data Availability

GEPIA (http://gepia.cancer-pku.cn/index.html) and Uniprot database(https://www.uniprot.org/) were used to predict the expression and function of TCP11. TCP11 protein distribution results from PSORT (https://psort.hgc.jp/). All data generated or analyzed during this study are included in this published article (and its supplementary information files).

## Abbreviations

GEPIA: Gene Expression Profiling Interactive Analysis
HE: Hematoxylin-Eosin
DMEM: Dulbecco’s Modified Eagle Medium
FBS: Fetal Bovine Serum
qRT-PCR: Real-time fluorescent quantitative polymerase chain reaction
ICC: Immunocytochemistry
HPV: Human papilloma virus
UCS: Uterine Carcinosarcoma
PARP: Poly ADP ribose polymerase
EMT: Epithelial-Mesenchymal Transition
ZO-1: Zonula occludens-1
CDK1: Cyclin-dependent kinases
cAMP: Cyclic adenosine monophosphate
PKA: Protein kinase A
