# Supplementary material for: Increased TCP11 gene expression can inhibit the proliferation, migration and promote apoptosis of cervical cancer cells
Source: BMC Cancer. 2023 Sep 11;23:853. doi: 10.1186/s12885-023-11129-1 (PMC10496356; doi:10.1186/s12885-023-11129-1)
Supplement: Supplementary file 1 — Supplementary Material 1 [file 12885_2023_11129_MOESM1_ESM.docx]

Table S1 Relationship between TCP11 gene expression and age and differentiation degree of patients

| Clinical features | N | The expression of TCP11 gene | | χ^2^value | P value |
| --- | --- | --- | --- | --- | --- |
|  |  | - | + |  |  |
| Age | 35 |  |  |  |  |
| <50 | 10 | 2 | 8 | 0.504 | 0.478 |
| >50 | 25 | 8 | 17 |  |  |
| Grade of pathology | 29 |  |  |  |  |
| Low differentiation | 5 | 1 | 4 | 0.885 | 0.642 |
| Moderate differentiation | 23 | 8 | 15 |  |  |
| High differentiation | 1 | 0 | 1 |  |  |

Table S2 Primer sequence

| Gene | Primer | Primer sequence (5´-3´) |
| --- | --- | --- |
| GAPDH | Forward | ATGCGTTGCTGGTACAACTG |
|  | Reverse | CACGCCTCAATGTCTTCTGA |
| TCP11 | Forward | GCAGTGTTTTGTTTGGCTCA |
|  | Reverse | GGCTTTGATGGATTTCCTGA |
| Ki67 | Forward | ACGCCTGGTTACTATCAAAAGG |
|  | Reverse | CAGACCCATTTACTTGTGTTGGA |
| CDK1 | Forward | AAACTACAGGTCAAGTGGTAGCC |
|  | Reverse | TCCTGCATAAGCACATCCTGA |
| CyclinB1 | Forward | GACTTTGCTTTTGTGACTGACA |
|  | Reverse | CCCAGACCAAAGTTTAAAGCTC |
| ZO-1 | Forward | CGGTCCTCTGAGCCTGTAAG |
|  | Reverse | GGATCTACATGCGACGACAA |
| E-cadherin | Forward | TTGAGAATGAGGTCGGTGCC |
|  | Reverse | CAGAATGCCCTCGTTGGTCT |


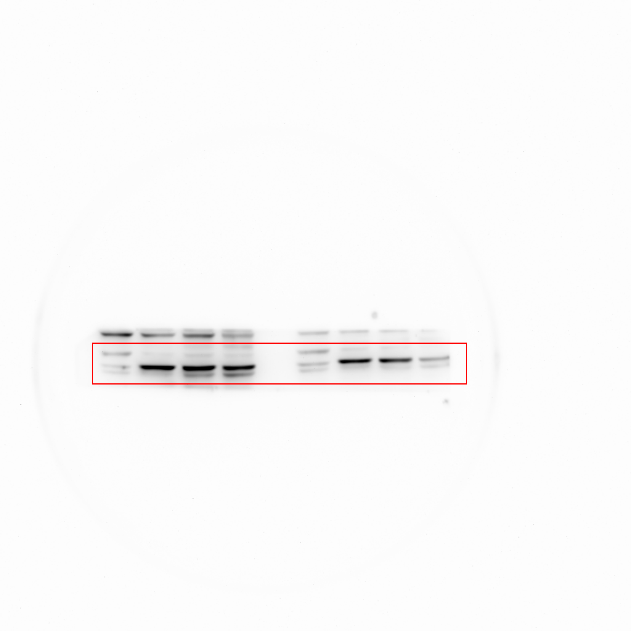

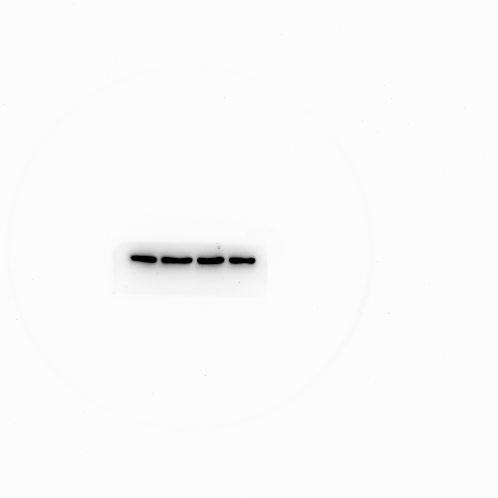


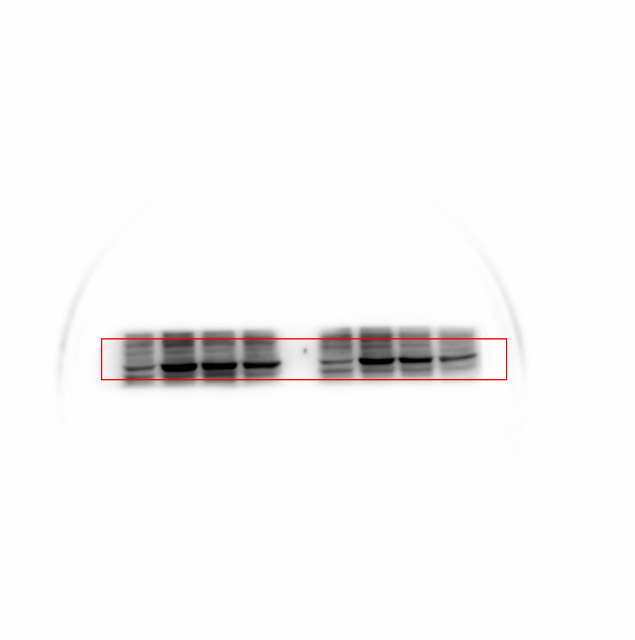

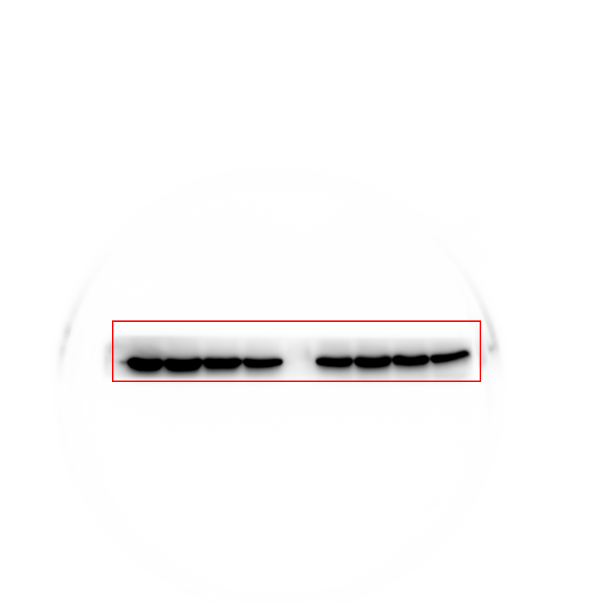


Figure S1. Full-length impression/gel of TCP11/GAPDH in Figure 1C.


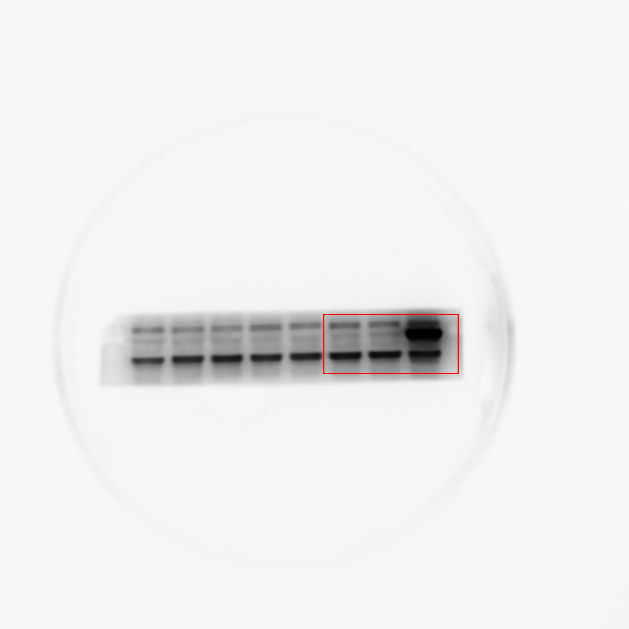

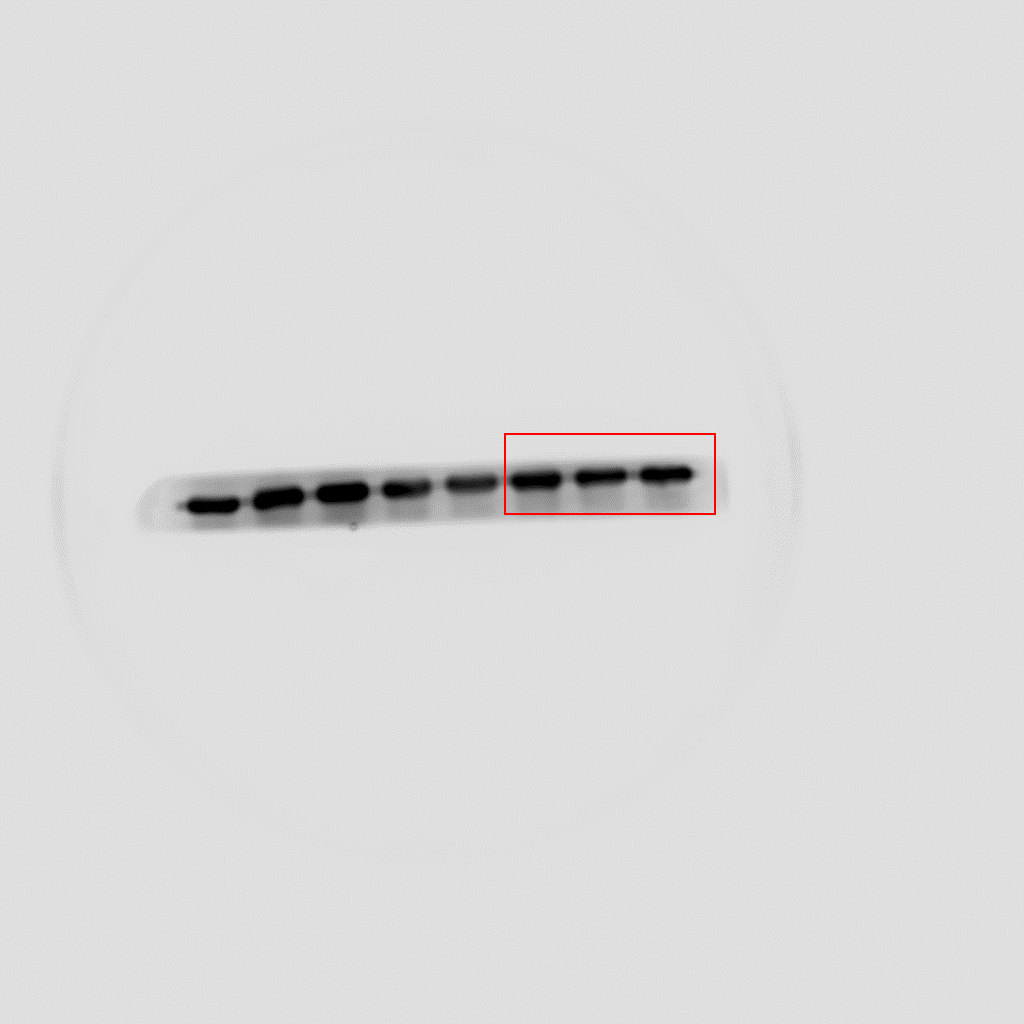


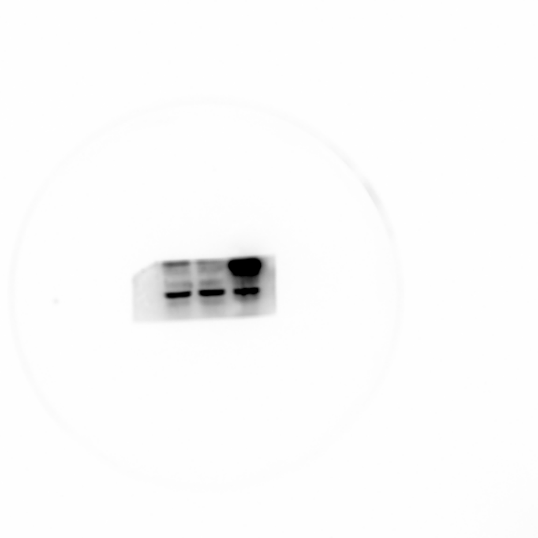

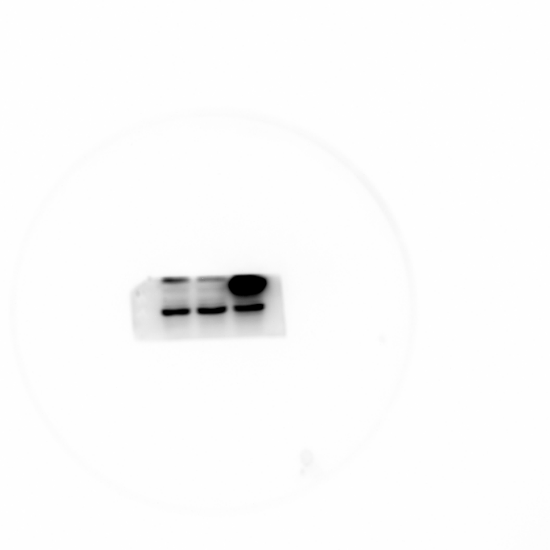


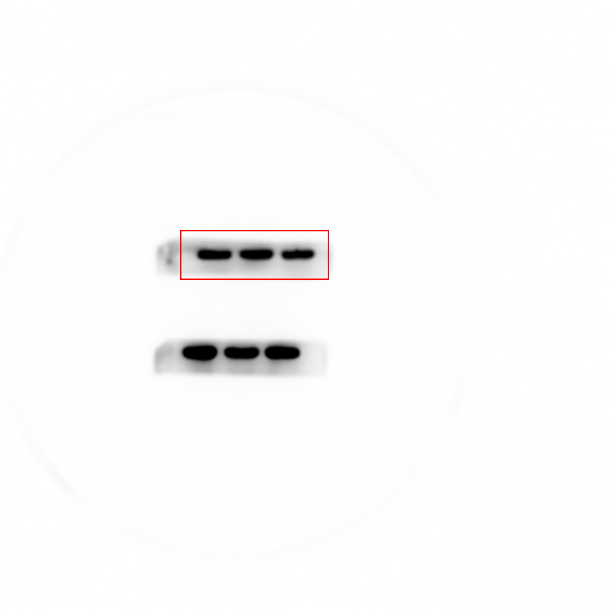

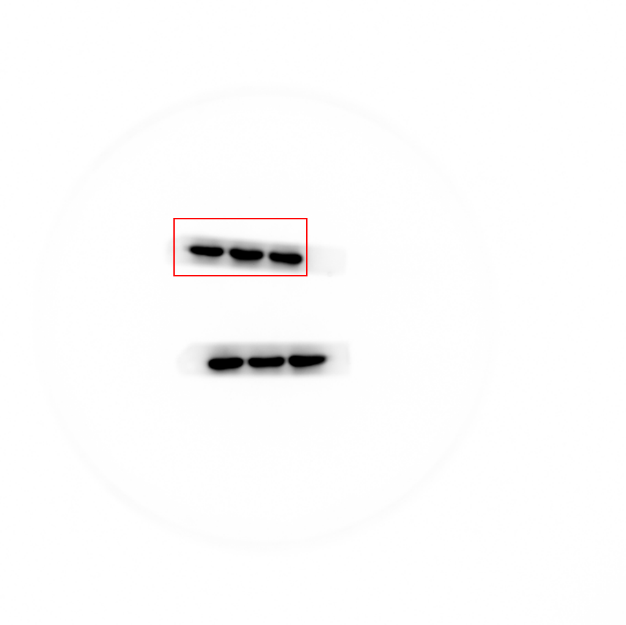


Figure S2. Full-length impression/gel of TCP11/GAPDH with HeLa cells in Figure 2A.


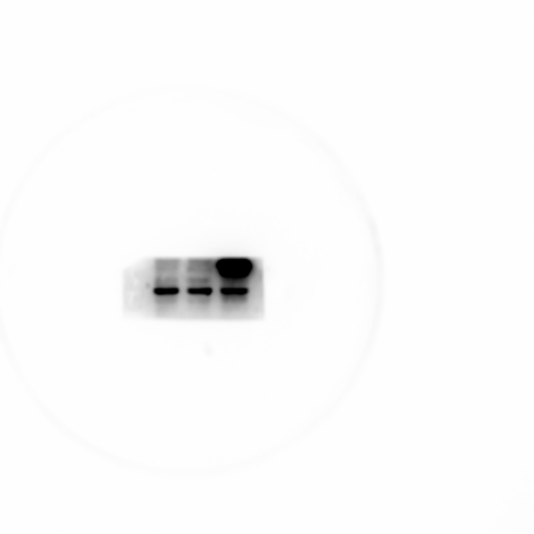

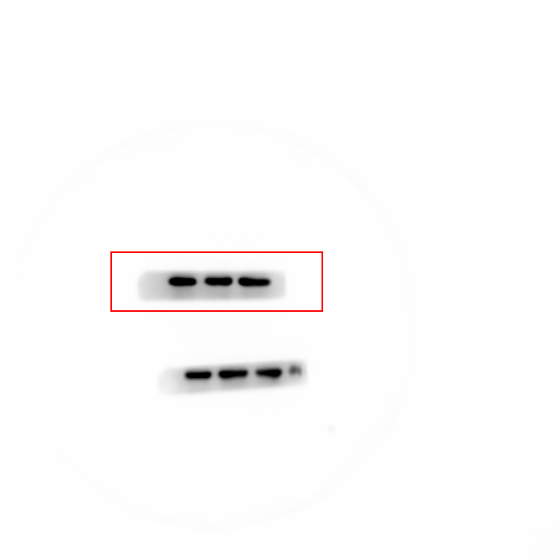


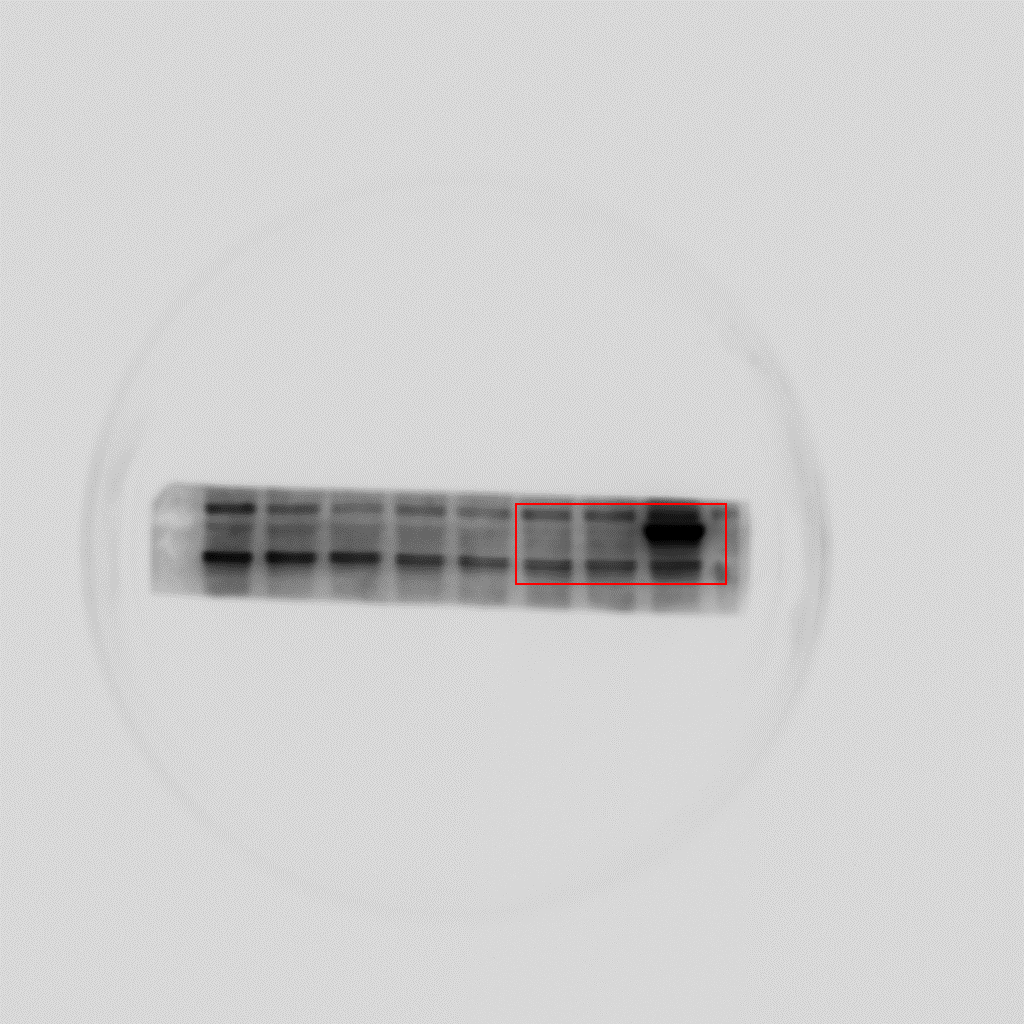

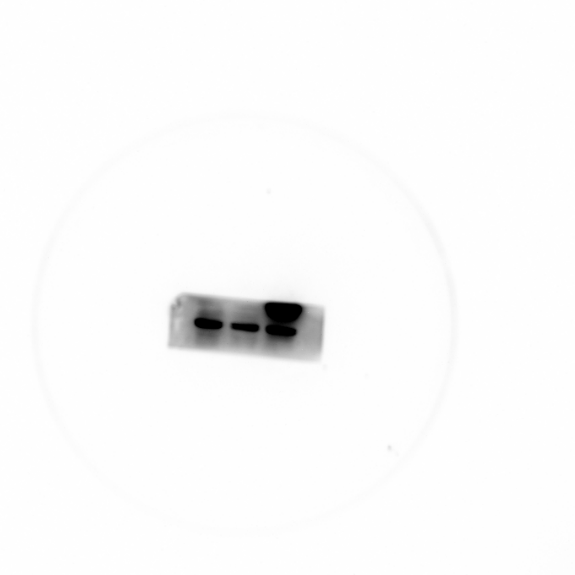


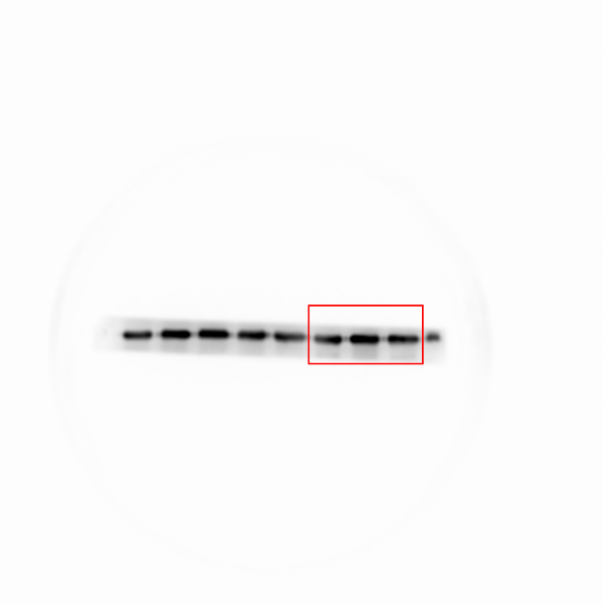

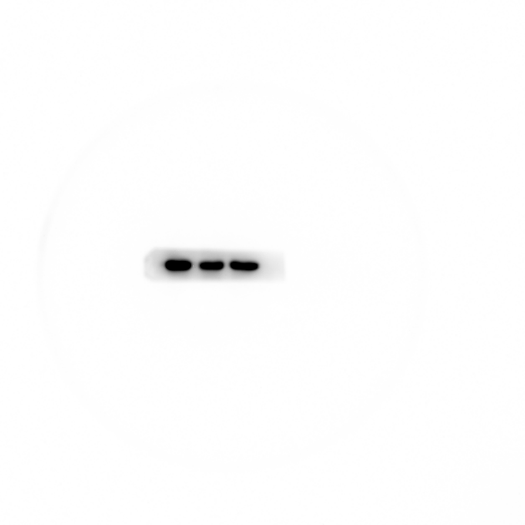


Figure S2. Full-length impression/gel of TCP11/GAPDH with SiHa cells in Figure 2A.


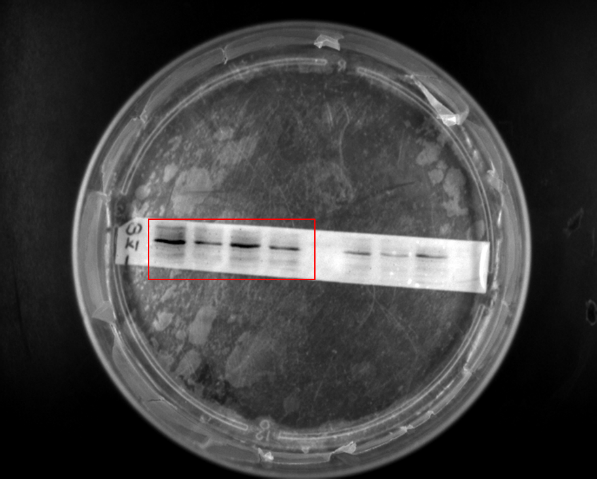

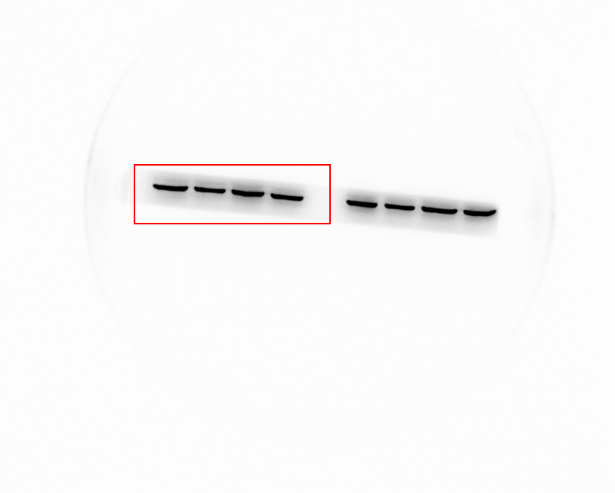


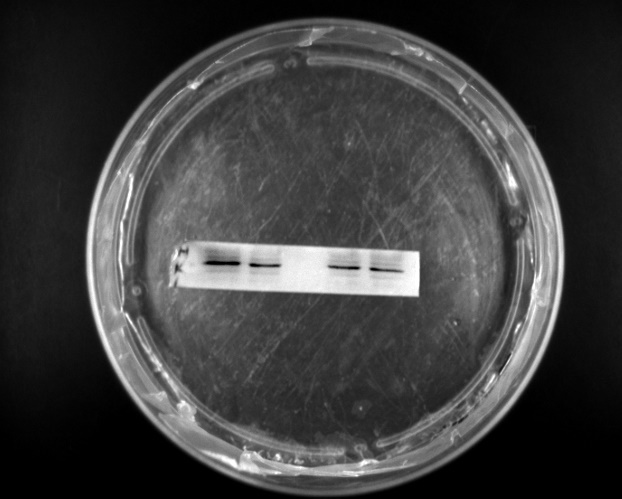

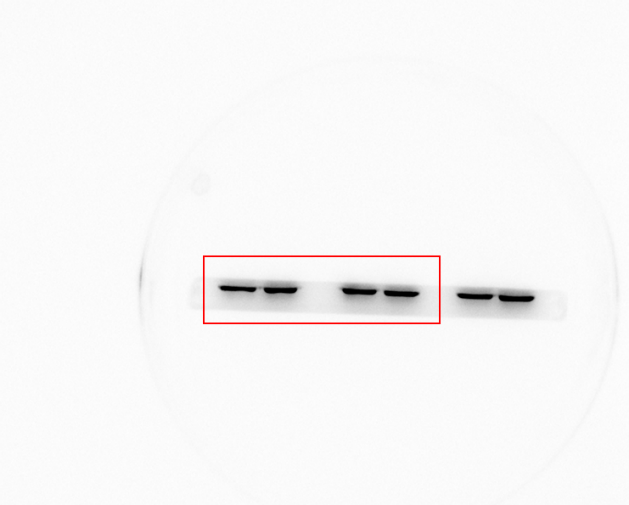


Figure S3A. Full-length imprinting/gel of CDK1/β-actin with HeLa cells in Figure 3C.


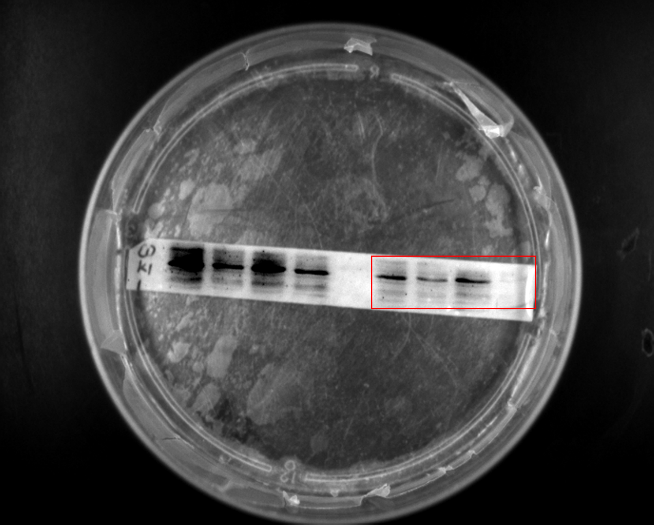

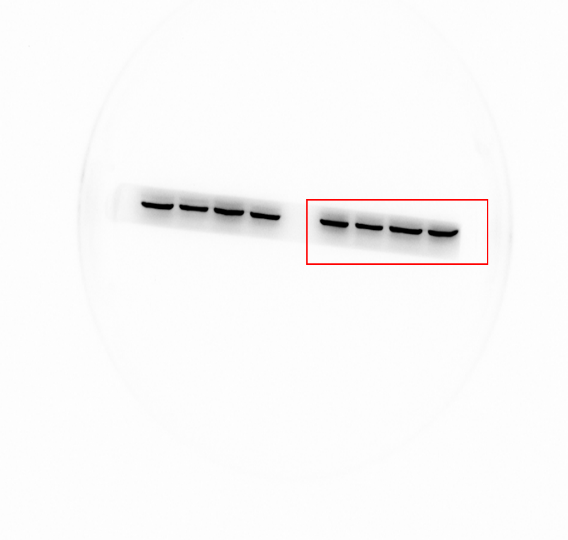


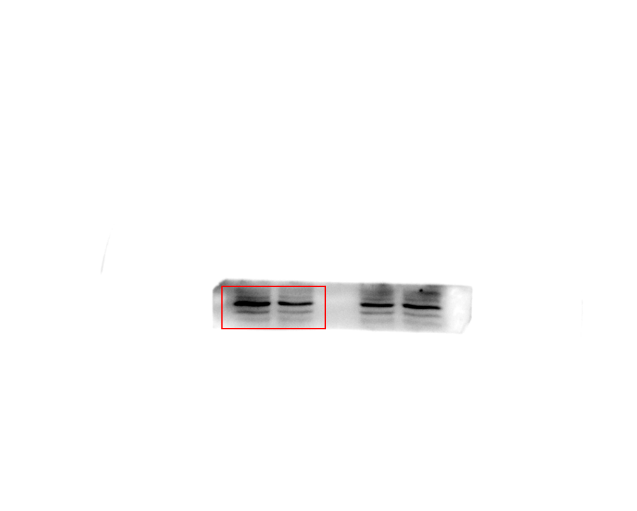

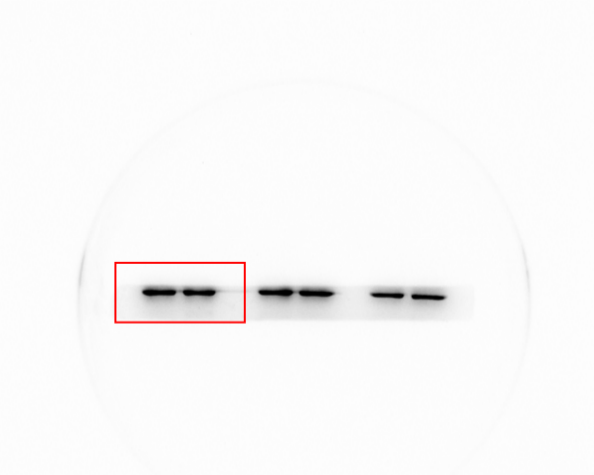


Figure S3A. Full-length imprinting/gel of CDK1/β-actin with SiHa cells in Figure 3C.


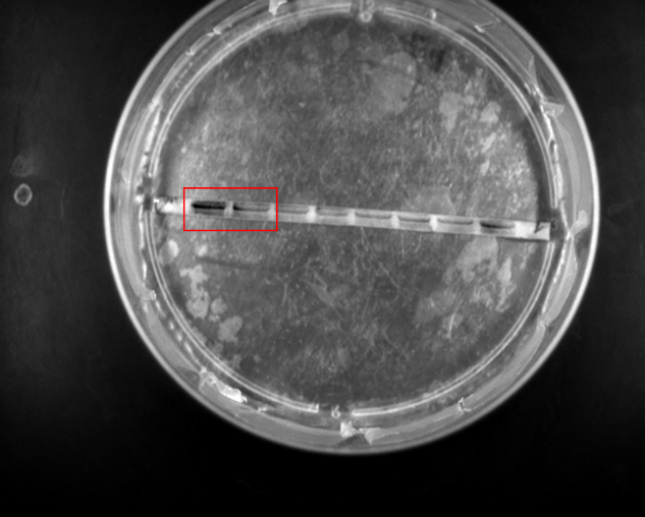

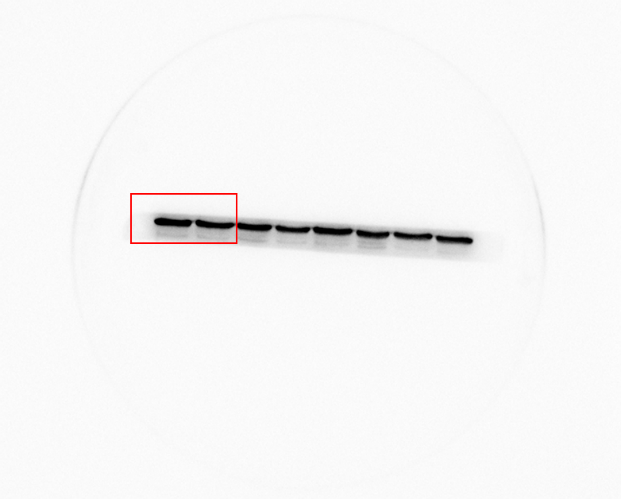


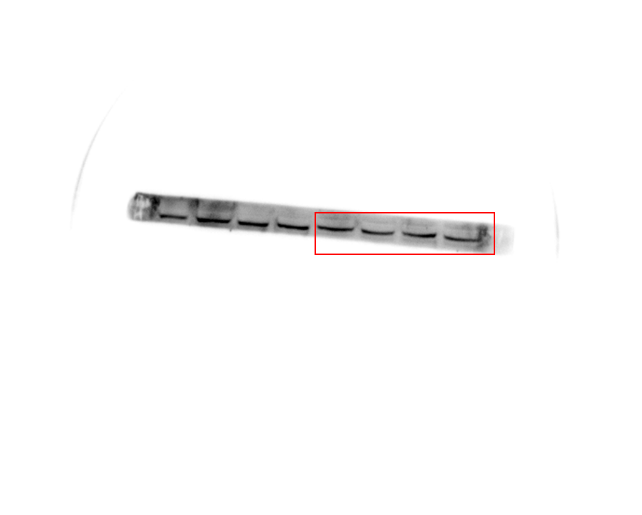

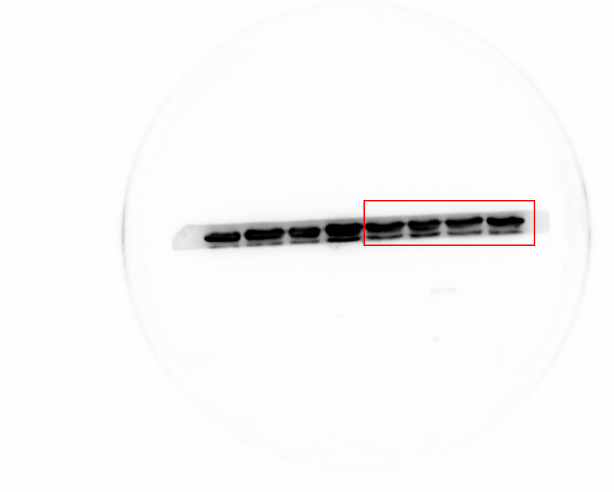


Figure S3B. Full-length imprinting/gel of CyclinB1/GAPDH in Hela cells in Figure 3C.


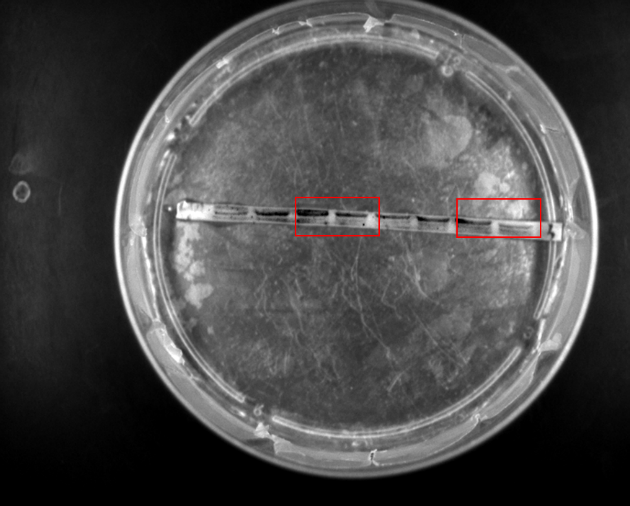

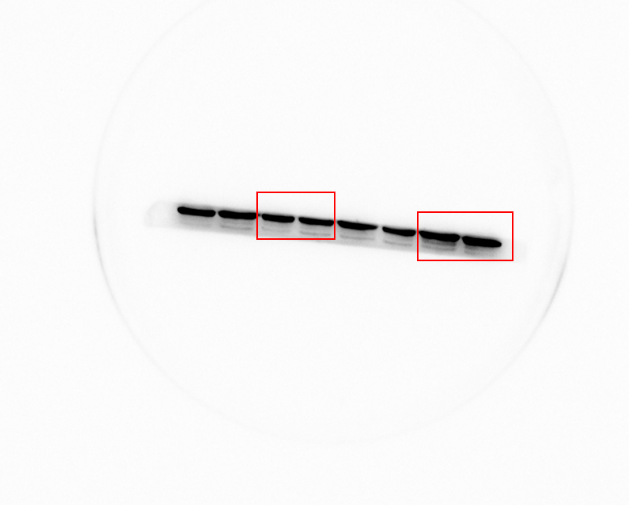


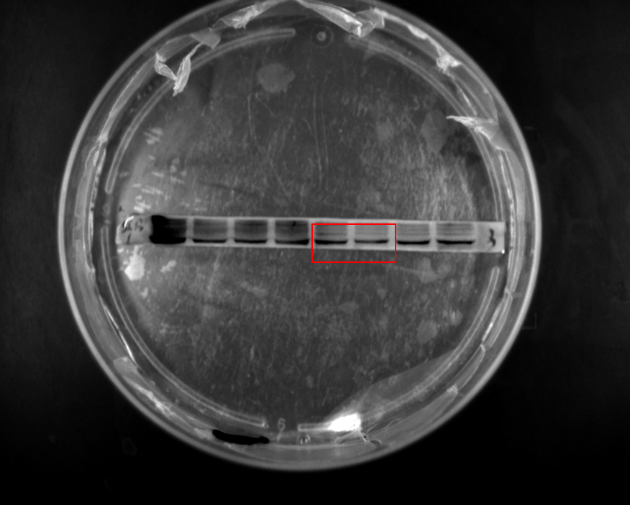

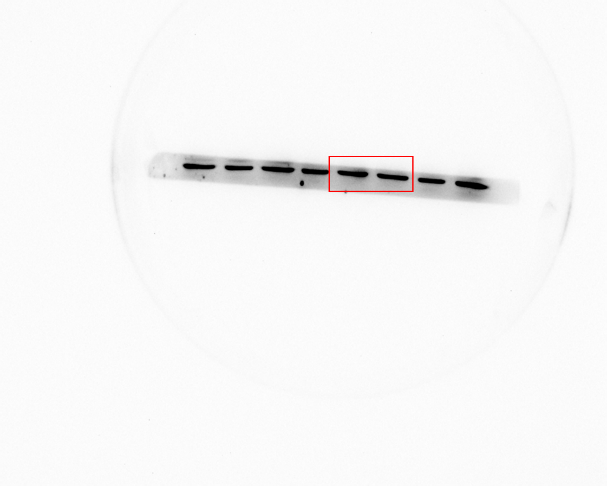


Figure S3C. Full-length imprinting/gel of CyclinB1/GAPDH in SiHa cells in Figure 3C.


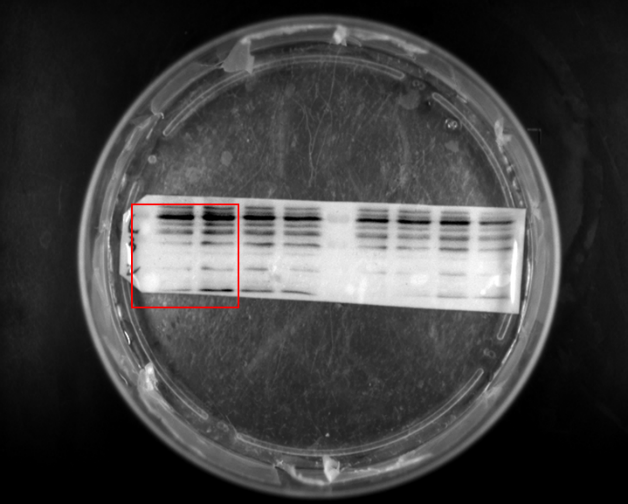

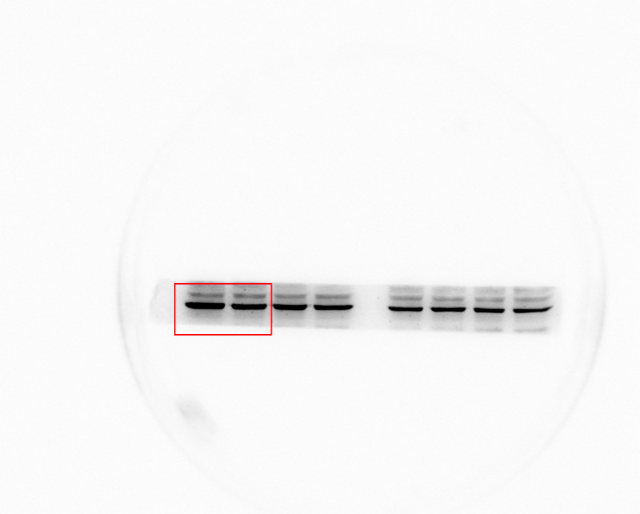


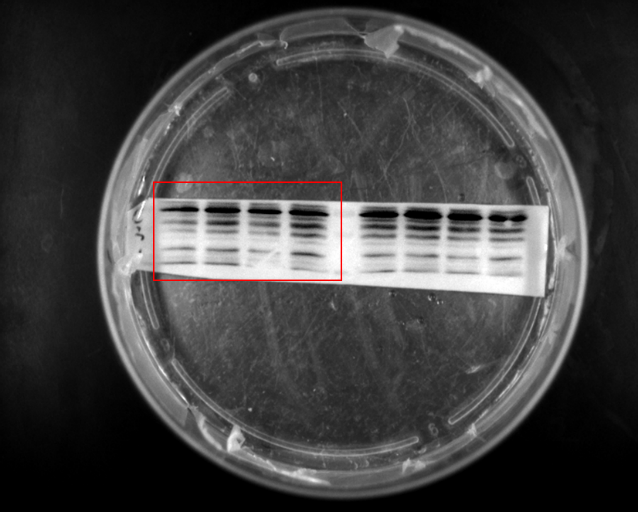

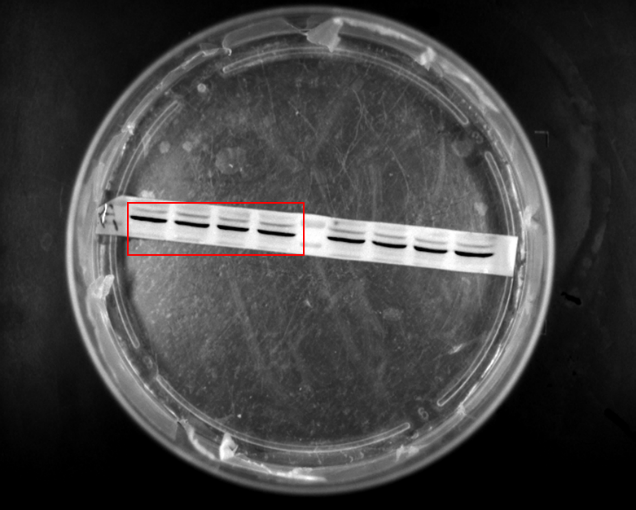


Figure S4A. Full-length imprinting/gel of caspase-3/cleaved-caspase-3 in HeLa cells in Figure 4C.


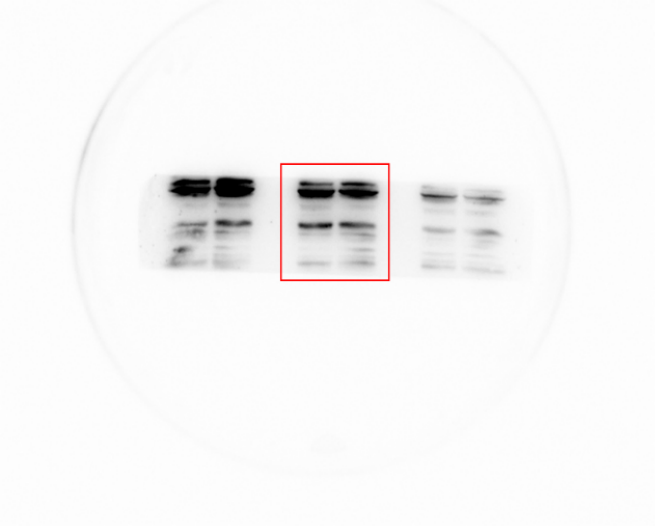

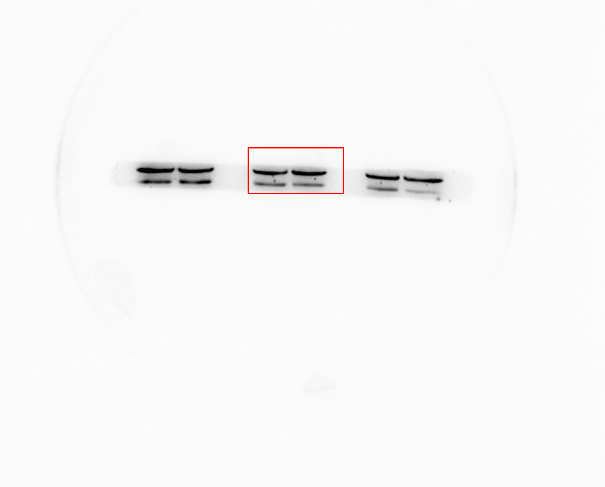

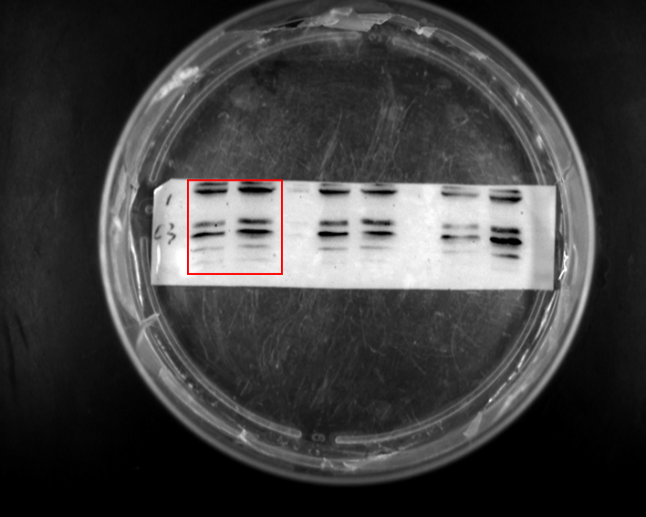

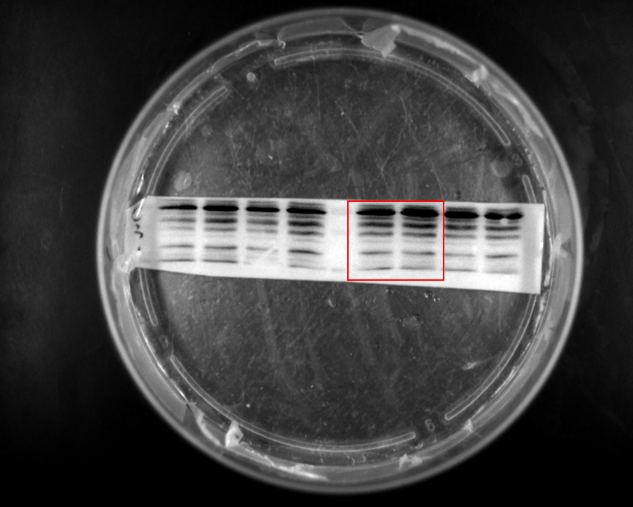


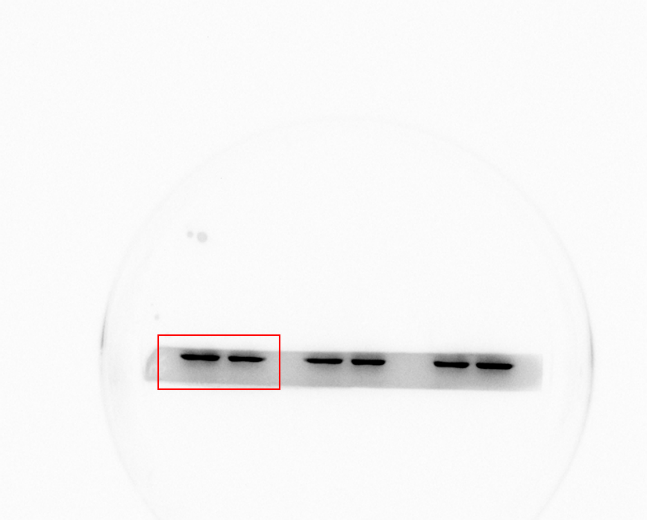

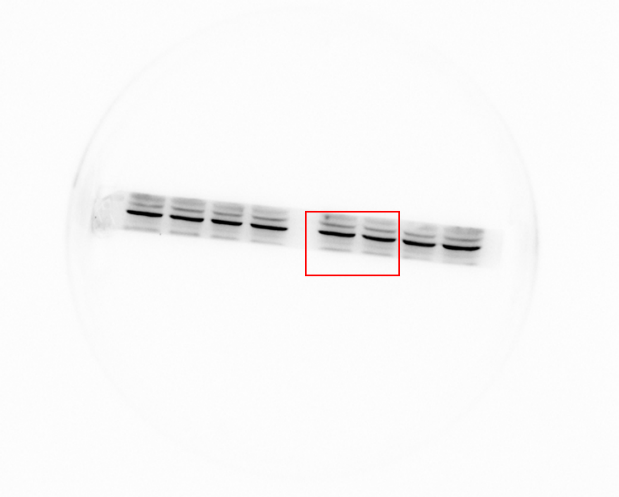


Figure S4B. Full-length imprinting/gel of caspase-3/cleaved-caspase-3/β-actin in SiHa cells in Figure 4C.


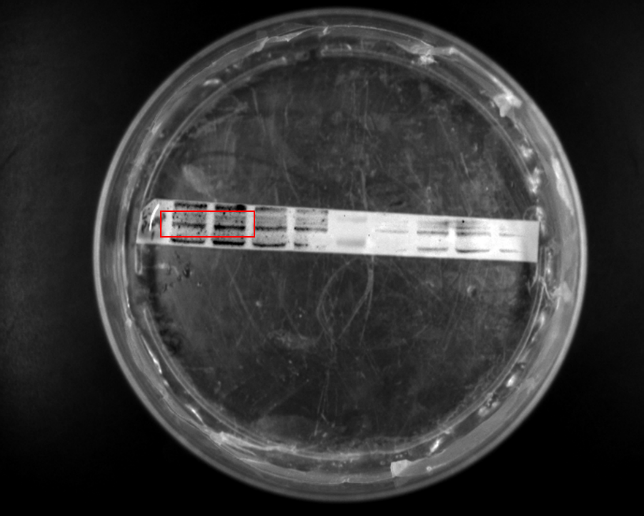

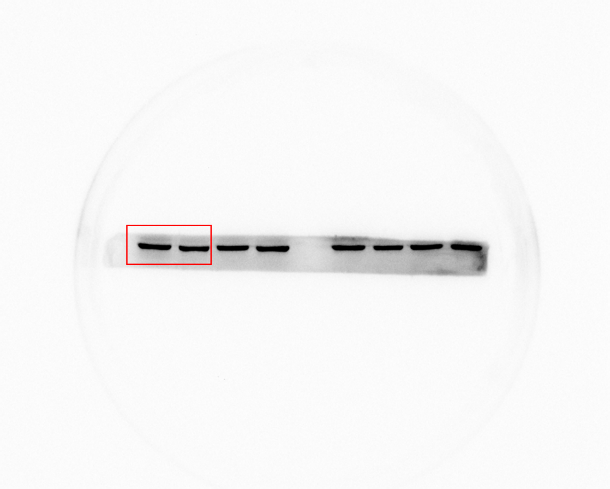


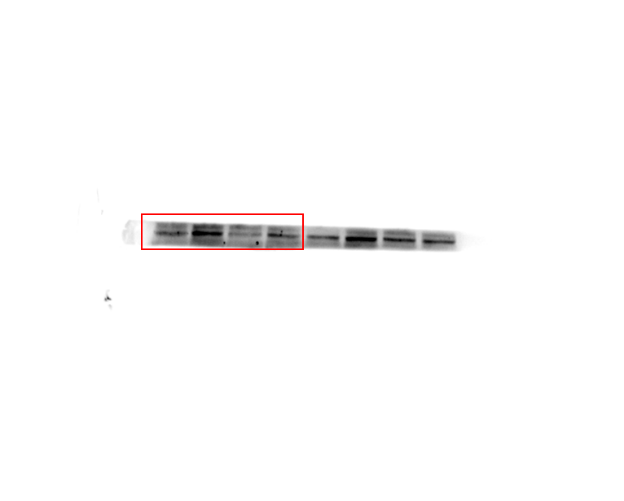

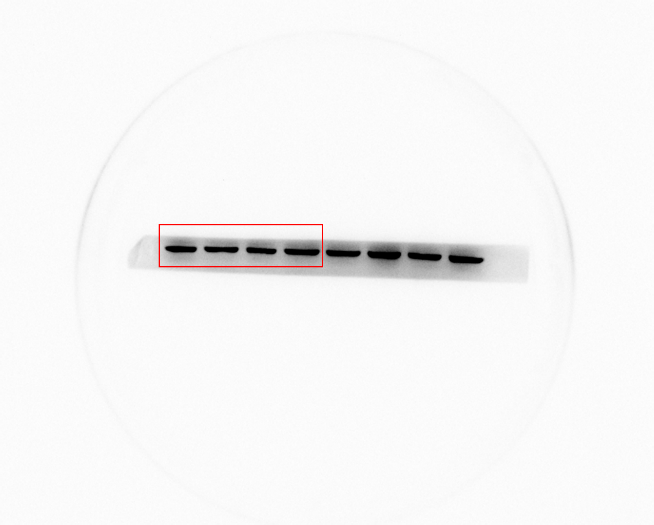


Figure S4C. Full-length imprinting/gel of Cleaved-PARP/β-actin with HeLa cells in Figure 4C.


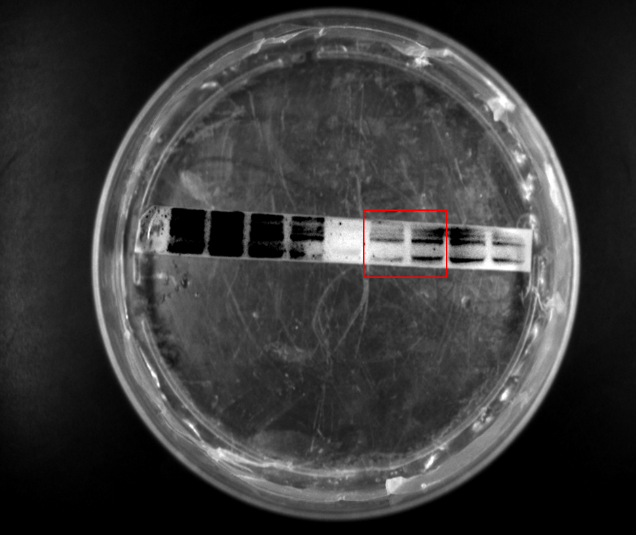

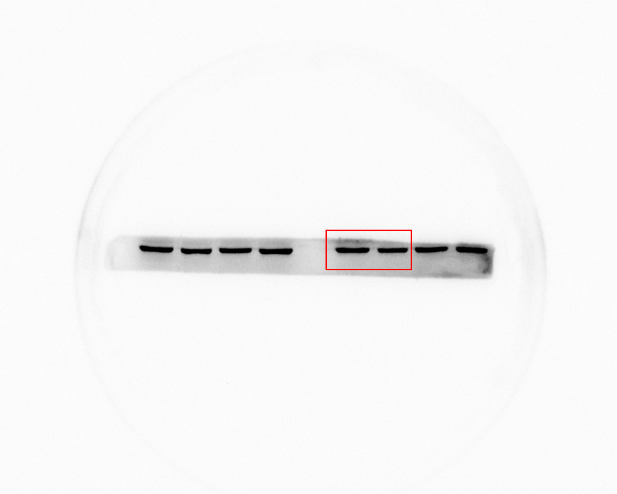


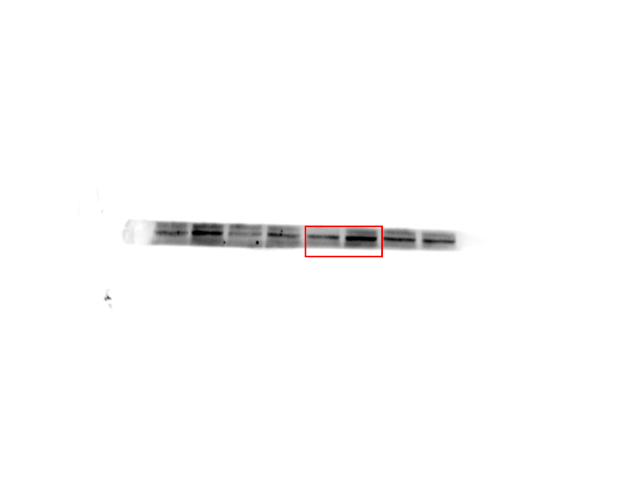

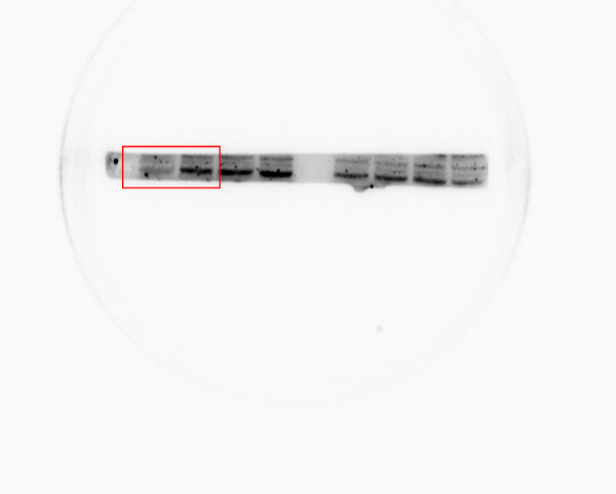


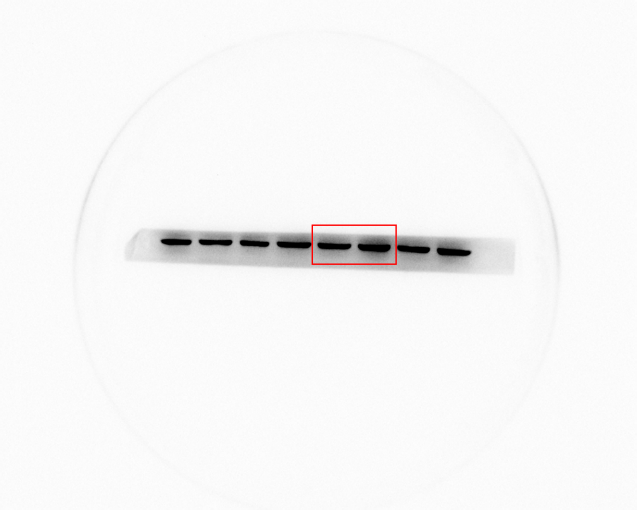

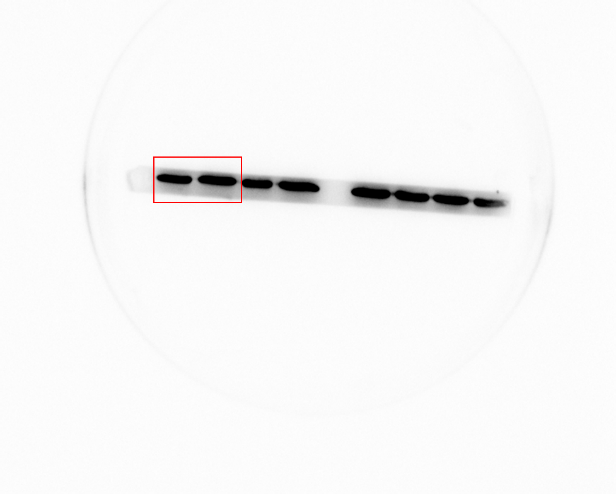


Figure S4C. Full-length imprinting/gel of Cleaved-PARP/β-actin with SiHa cells in Figure 4C.


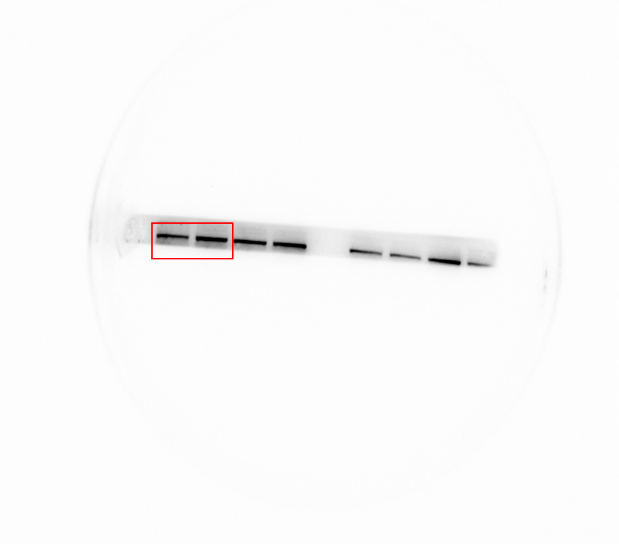

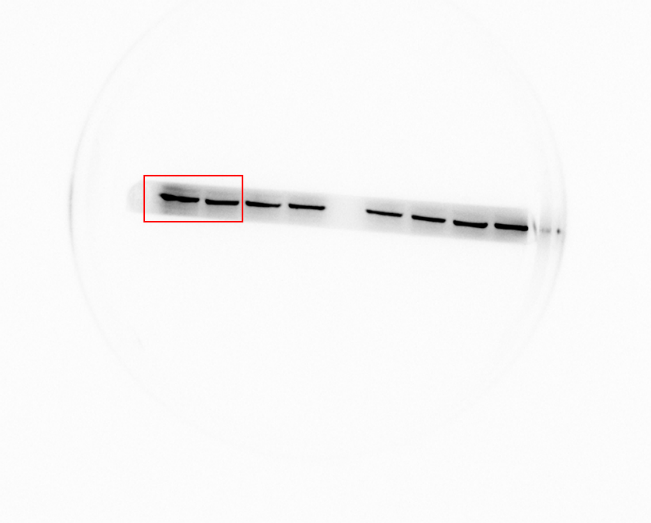


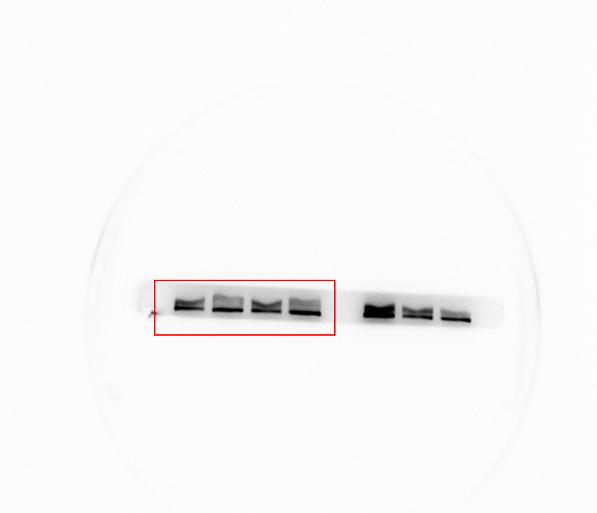

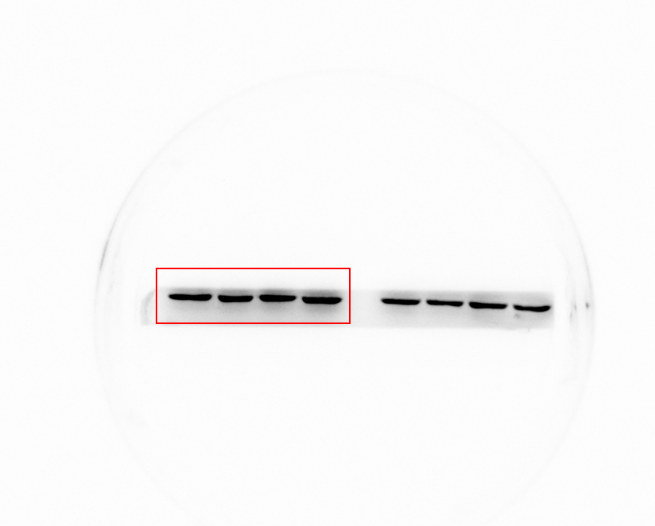


Figure S5A. Full-length imprinting/gel of E-cadherin/β-actin in HeLa cells in Figure 5E.


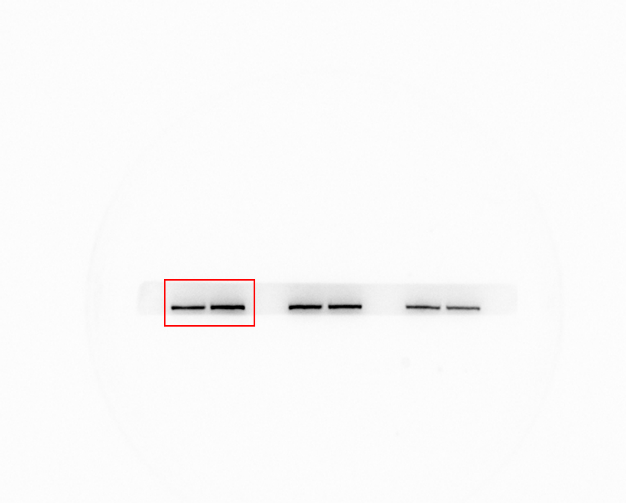

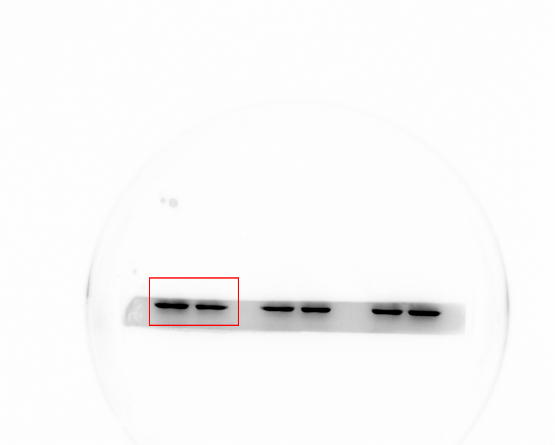


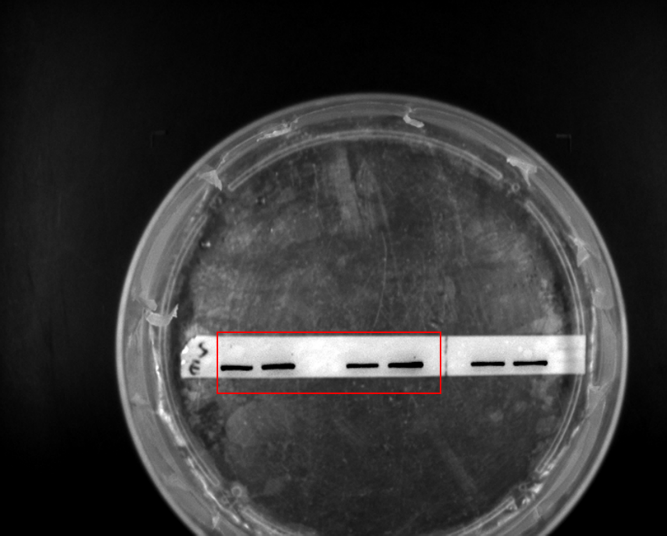

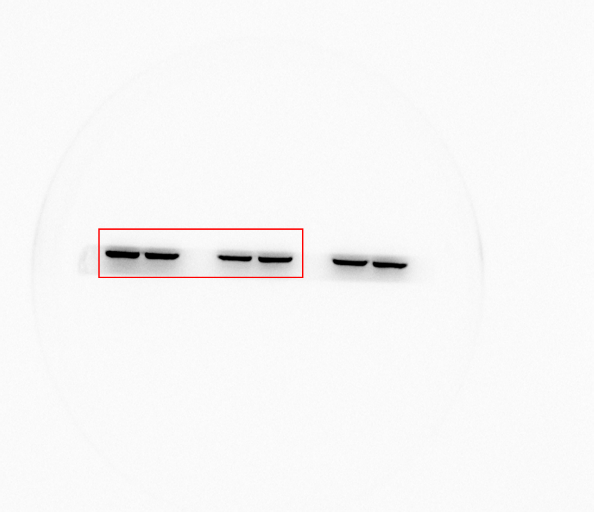


Figure S5B. Full-length imprinting/gel of E-cadherin/β-actin in SiHa cells in Figure 5E.


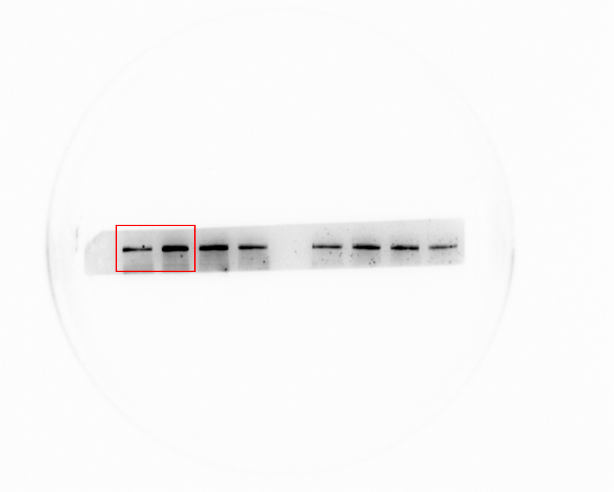

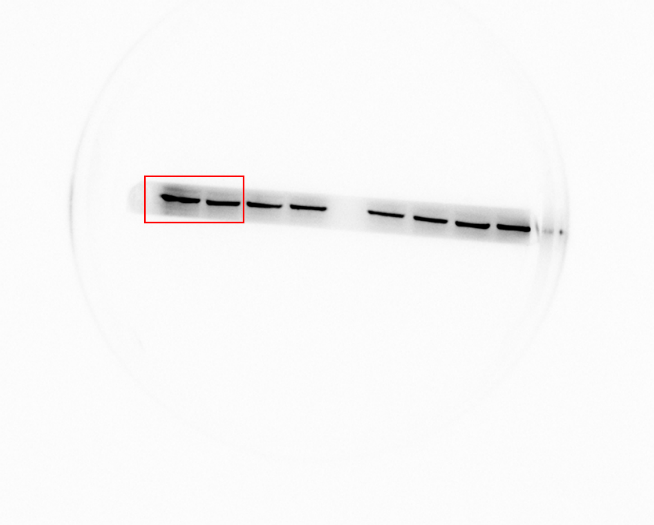

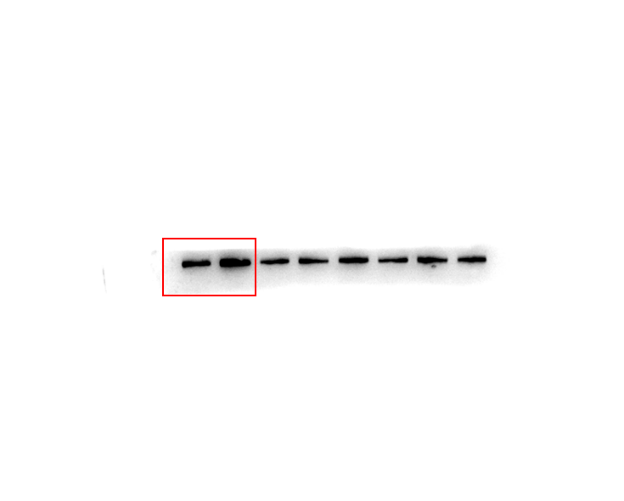

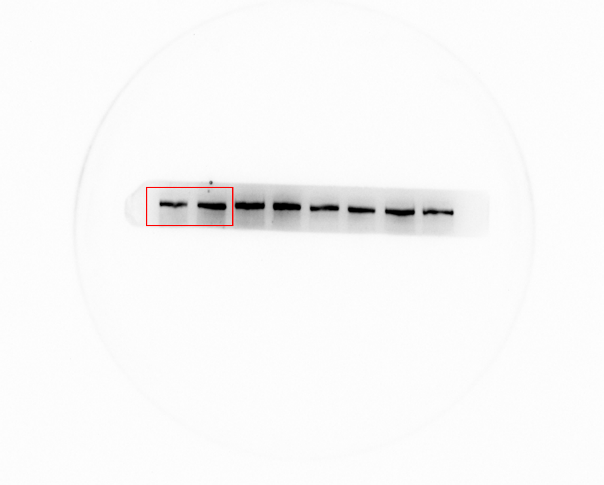


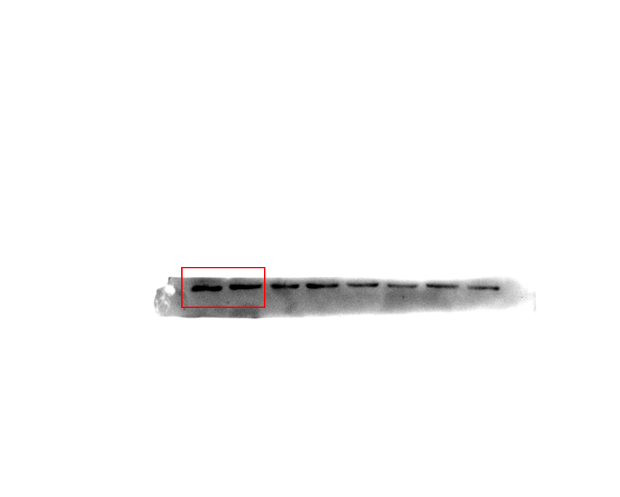

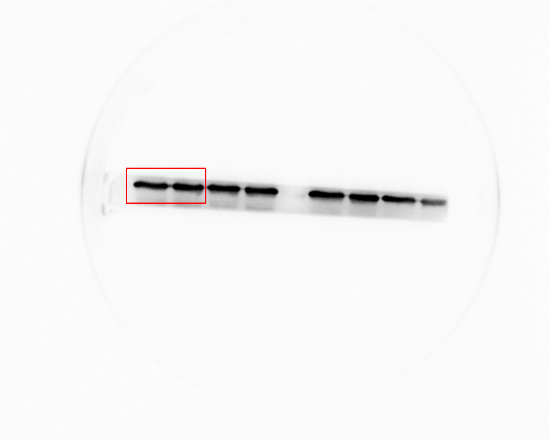


Figure S5C. Full-length imprinting/gel of ZO-1/β-actin with HeLa cells in Figure 5E.


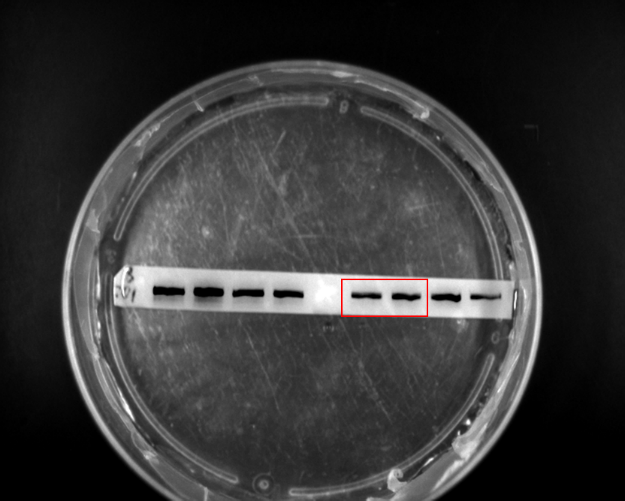

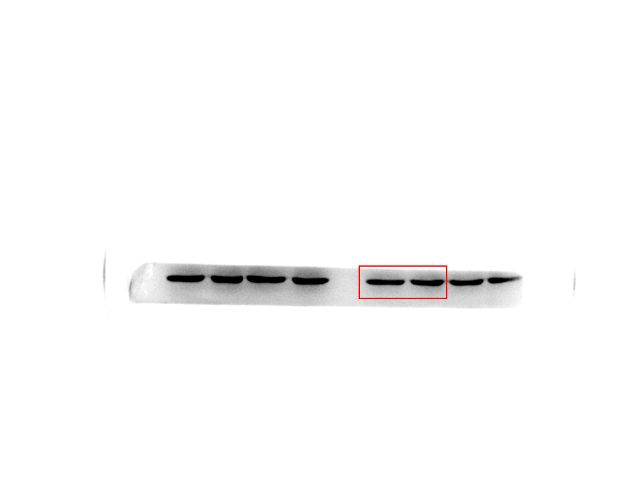


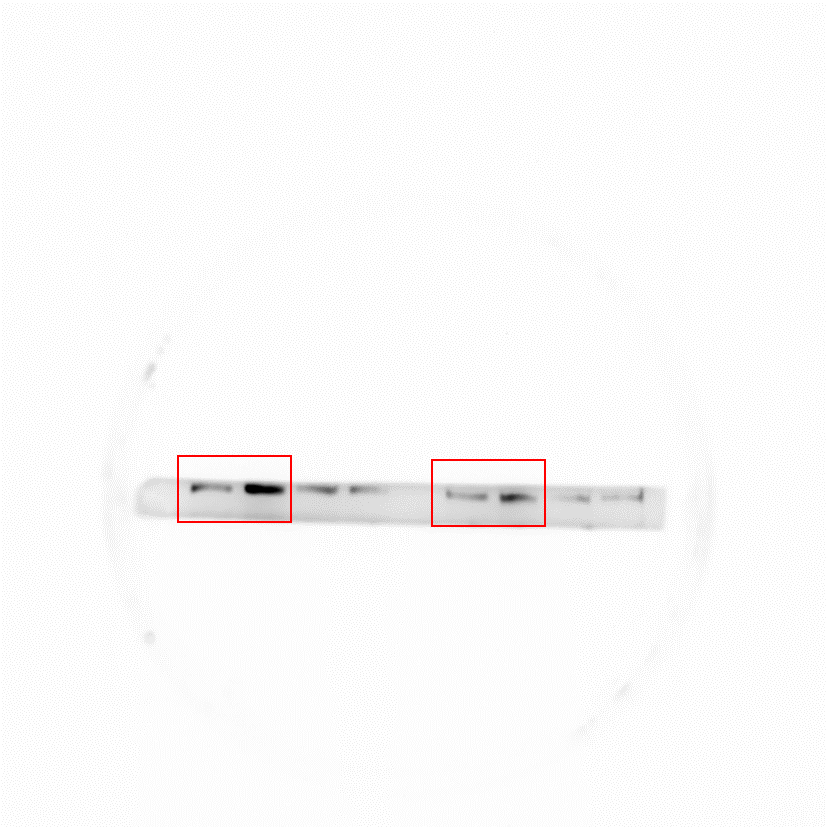

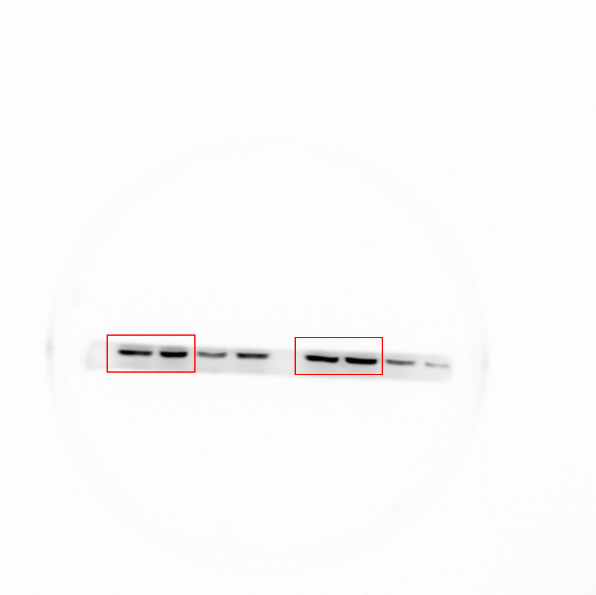


Figure S5C. Full-length imprinting/gel of ZO-1/β-actin with SiHa cells in Figure 5E.


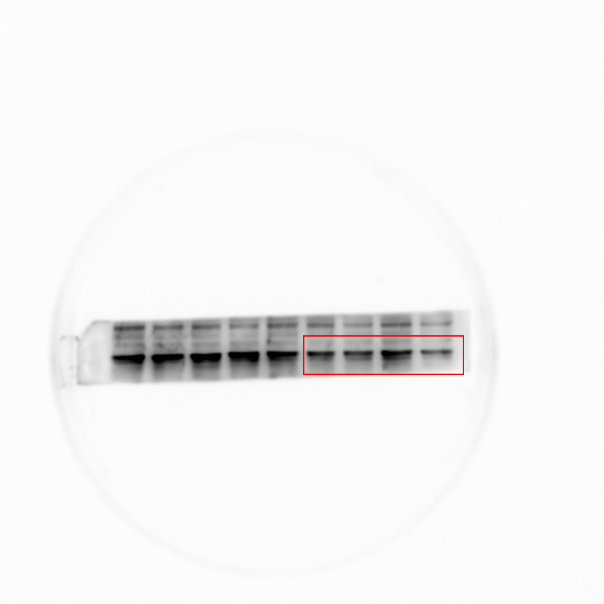

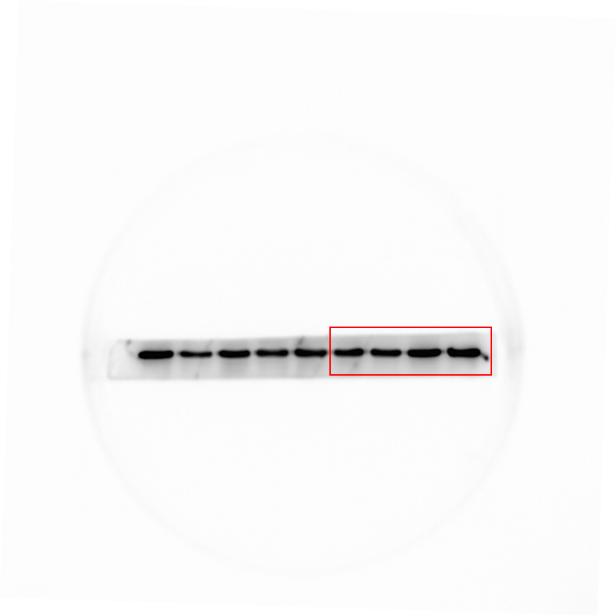


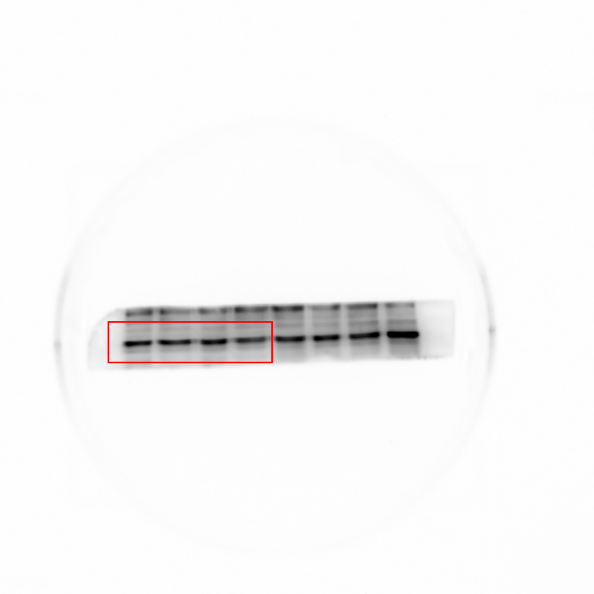

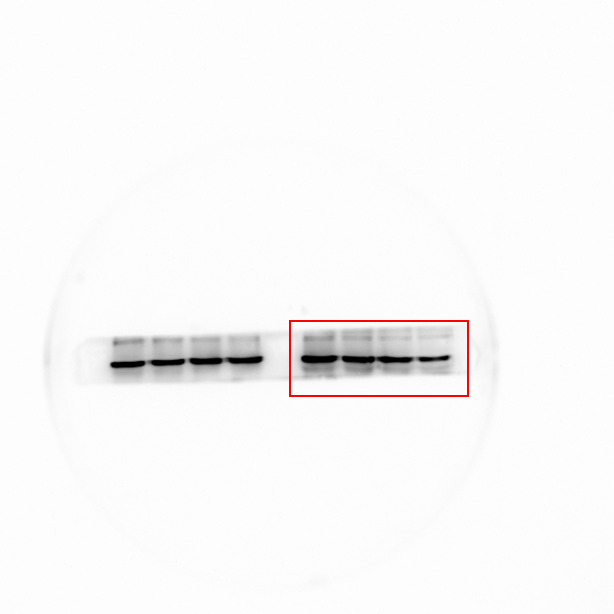

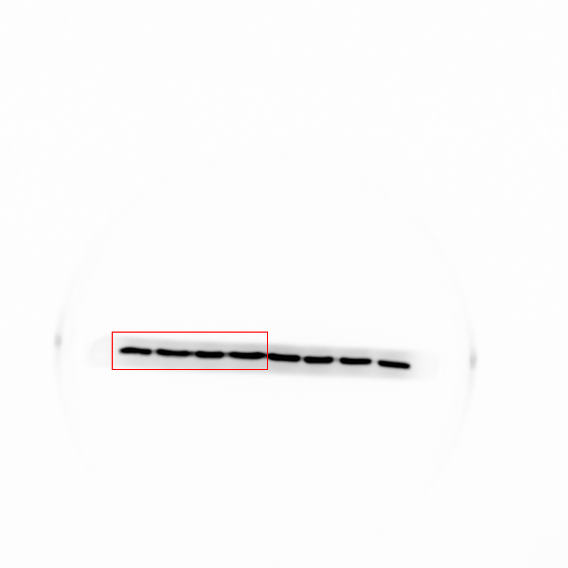

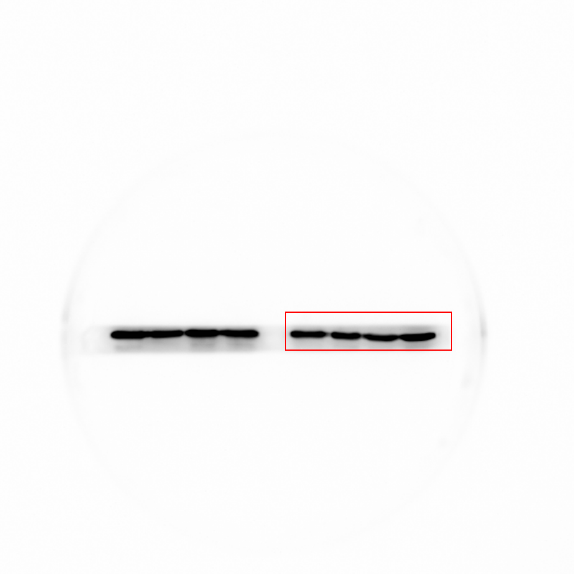


Figure S6. Full-length imprinting/gel of TCP11/GAPDH in HeLa cells in Figure 6A.
